# Selective Protonation of Catalytic Dyad for γ-Secretase-Mediated Hydrolysis Revealed by Multiscale Simulations

**DOI:** 10.1021/acs.jpcb.4c04085

**Published:** 2024-11-07

**Authors:** Bohua Wu, Shu Li, Wei Han

**Affiliations:** † State Key Laboratory of Chemical Oncogenomics, Guangdong Provincial Key Laboratory of Chemical Genomics, School of Chemical Biology and Biotechnology, Peking University Shenzhen Graduate School, Shenzhen 518055, China; ‡ Centre for Artificial Intelligence Driven Drug Discovery, Faculty of Applied Sciences, Macao Polytechnic University, Macao 999078, China; § Department of Chemistry, Faculty of Science, Hong Kong Baptist University, Hong Kong SAR 999077, China; ∥ Institute of Chemical Biology, Shenzhen Bay Laboratory, Shenzhen 518132, China

## Abstract

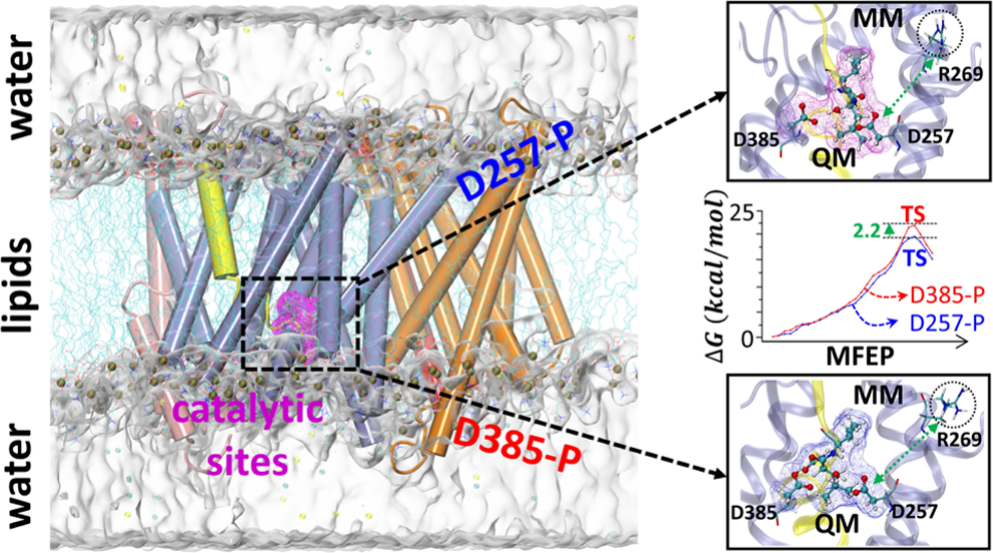

γ-Secretase plays a crucial role in producing disease-related amyloid-β proteins by cleaving the amyloid precursor protein (APP). The enzyme employs its catalytic dyad containing two aspartates (Asp257 and Asp385) to hydrolyze the substrate by a general acid–base catalytic mechanism, necessitating monoprotonation of the two aspartates for efficient hydrolysis. However, the precise protonation states of the aspartates remain uncertain. In this study, we employed a multiscale computational approach to investigate the dependence of the catalytic efficiency of γ-secretase on the protonation states of its catalytic dyad. Over 200 ms unbiased atomistic simulations of the substrate-enzyme complex reveal diverse orientations of the scissile bond of the bound substrate and accessible structural ensembles of the catalytic dyad with Asp257-Asp385 distances fluctuating between 4 and 10 Å. With a quantum mechanics/molecular mechanics (QM/MM) approach accelerated by enhanced sampling techniques, we find that the first step of the hydrolysis reaction, i.e., the formation of a gem-diol intermediate, experiences a higher reaction barrier by ∼2 kcal/mol when Asp385 is protonated. Furthermore, we find that Arg269 of the enzyme is most likely responsible for this preference of the protonation state: its basic side chain is spatially close to that of Asp257 and specifically stabilizes the transition state electrostatically when Asp257 is protonated. Collectively, our study suggests that Asp257 is likely the favored protonation site for APP cleavage by γ-secretase.

## 1 Introduction

**Figure 1:**
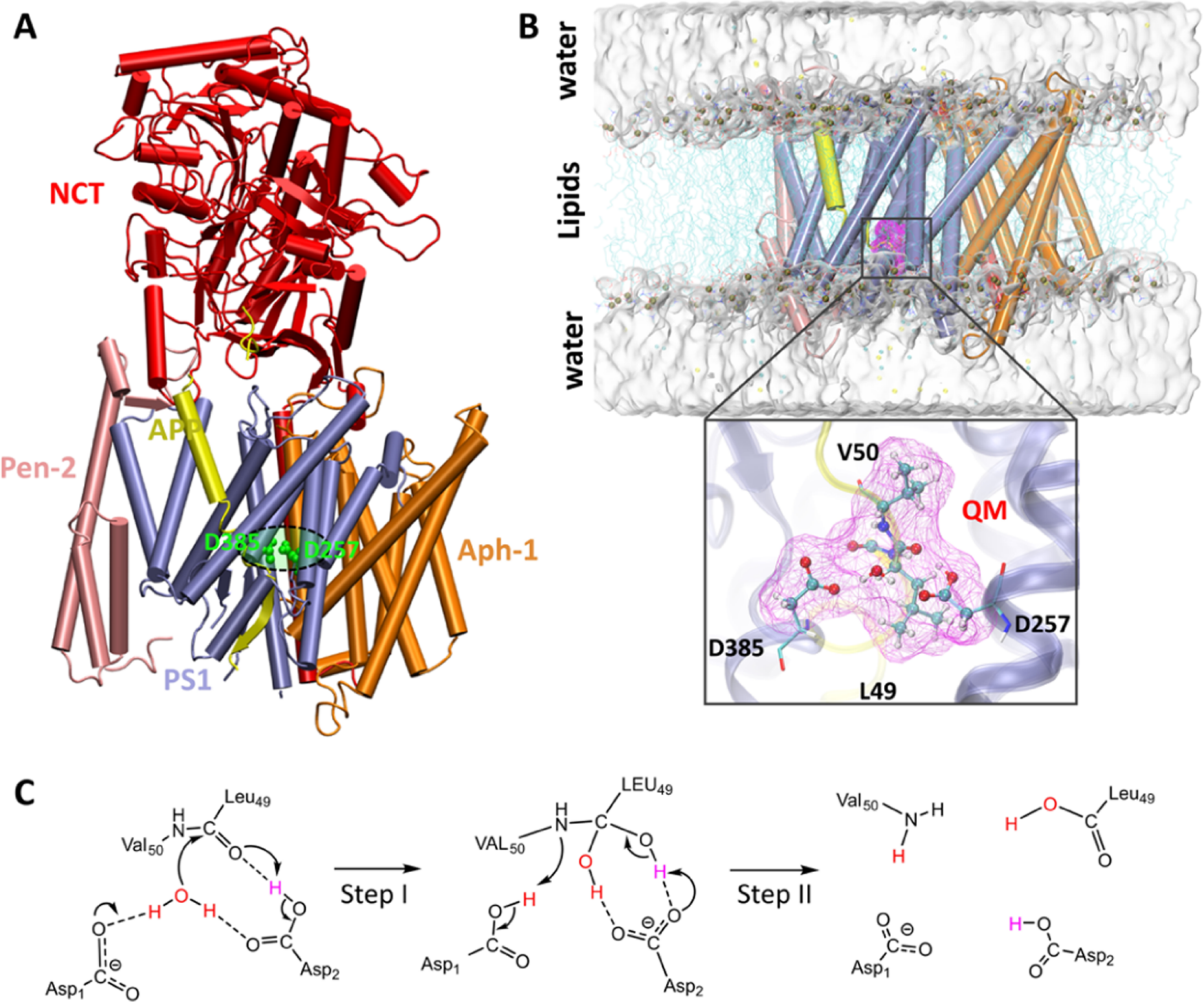
(A) Structure of γ-secretase in complex with APP (PDB entry 6IYC). The enzyme consists of four components: NCT (red), Aph-1 (orange), Pen-2 (pink), and PS1 (purple). The two catalytic residues (D257 and D385) of PS1 are shown as green beads. (B) Schematic representation of the QM/MM treatment of the APP-γ-secretase complex. The QM region of the system is shown with the wireframe. (C) General acid/base mechanism of the aspartyl protease family.

γ-Secretase is a membrane-embedded aspartyl protease complex composed of four subunits, nicastrin (NCT), anterior pharynx-defective 1 (Aph-1), presenilin enhancer 2 (Pen-2), and presenilin-1 (PS1) (Figure 1A).^1^ PS1 is the catalytic subunit of γ-secretase, which carries out intramembrane proteolysis of more than 150 different substrates, including particularly amyloid precursor protein (APP) closely related to Alzheimer’s disease.^2,3^ Proteolysis of APP by either α-secretase or β-secretase results in the production of C-terminal fragments of either 83-amino acids (C83) or 99-amino acids (C99), respectively.^4,5^ Initial endoproteolysis of C99 at the ε cleavage site by γ-secretase generates Aβ49 and Aβ48 fragments and corresponding APP intracellular domain (AICD50–99 and AICD49–99).^6^ The resulting Aβ49 and Aβ48 are then trimmed stepwisely every 3–4 amino acids through carboxypeptidase activity of γ-secretase, leading to the production of two main Aβ isomers, Aβ40 and Aβ42, the ratio of which is correlated with the pathogenesis of Alzheimer’s disease (AD).^7,8^

The ε cleavage of APP is catalyzed by the aspartate dyad (Asp257 and Asp385) of γ-secretase by a general acid/base hydrolysis mechanism.^9−13^ This mechanism consists of two steps (Figure 1C). In step I, one of the two aspartates acquires a proton, and the substrate presents its scissile peptide bond to the catalytic dyad, forming a tetrahedral gem-diol intermediate. The deprotonated aspartate acts as a general base and activates a nearby water molecule by accepting its proton; the protonated aspartate acts as a general acid by donating the proton to the oxygen atom of the scissile bond. A nucleophilic attack takes place between the activated water and the scissile bond, giving rise to a gem-diol intermediate. In step II, the aspartate that activates water gives up its proton to the nitrogen atom of the scissile peptide bond, while the other aspartate obtains a proton from the gem-diol, resulting in the cleavage of the peptide bond. Multiple computational studies of catalytic mechanisms of other aspartate proteases have revealed that the formation of the gem-diol intermediates (step I) is the rate-limiting step for the cleavage of peptide bonds.^9,14^ As for γ-secretase, a comprehensive quantum mechanical (QM) calculation with a minimal model of the active site and the substrate segment has also confirmed that step I is the rate-limiting step for the cleavage of Val40–Ile41 and Ala42–Thr43 peptide bonds of APP by PS1.^10^

Despite the importance of the formation of gem-diol intermediates for the overall reaction, mechanistic details regarding this critical step remain elusive. In particular, the protonation states of the catalytic dyad at the onset of the hydrolysis may determine how the reaction could proceed.^15−17^ It is important to know whether and which aspartate in the catalytic dyad is a preferred site for protonation. There have been numerous computational studies conducted to address this question from the perspective of the thermodynamic stability of protonation states. However, the results remain somewhat controversial. Aguayo-Ortiz et al. proposed that Asp257 should be protonated through MD simulations and p*K*_a_ calculation with PROPKA.^18^ With Gaussian accelerated molecular dynamics (GAMD) techniques, Bhattarai et al. found that γ-secretase can access more readily multiple conformations of γ-secretase, including inhibited, intermediate, inactive, and active ones where the protonated Asp257 was favorable.^19^ In contrast, another study with MD simulations and p*K*_a_ calculation supported the protonation of Asp385.^20^ Furthermore, through more rigorous constant pH simulations, Guzmán-Ocampo et al. revealed that Asp385 is more likely than Asp257 to be protonated.^21^

Nonetheless, apart from the stability difference in the protonation states, γ-secretase in different protonation states may also need to overcome distinct reaction barriers. Thus, it may not be possible to assess the preferred protonation state of the catalytic dyad unless the difference in reaction barriers between different protonation states could be determined, which necessitates a computational investigation of the catalytic mechanism of γ-secretase in different protonation states. Despite numerous simulations of structures and dynamics of γ-secretase with classical molecular mechanics,^19,20,22−26^ the actual reaction is rarely studied computationally except for the QM calculation of model systems where the full realistic environment of this intramembrane enzyme crucial for understanding the effect of protonation states was considered little.^10^ One of the major hurdles for a more realistic modeling of this reaction is attributed to the lack of a structural model for active conformations of γ-secretase. Although a series of experimental structures of γ-secretase were solved by cryo-EM,^27−30^ these crystal structures are nonactive conformation owing to the absence of substrate or the mutation of catalytic residue to prevent substrate cleavage. In the active conformation, the two catalytic aspartates (Asp257 and Asp385) are at a suitable distance so that a nucleophilic water molecule can be recruited for the proteolytic reaction through water-bridged hydrogen bonding with the two aspartates.^19,31^

In this study, we employed extensive unbiased MD simulations and a hybrid quantum mechanics/molecular mechanics (QM/MM) MD approach to investigate the nucleophilic attack step of the hydrolysis reaction mechanism of APP catalyzed by γ-secretase in different protonation states of the catalytic dyad. We show here that the active conformations are accessible through tens of microseconds of unbiased simulations, starting from experimental structures of the substrate-enzyme complex. With these active conformations, our QM/MM studies reveal that the catalytic capability of γ-secretase is significantly dependent on the initial protonation states of the dyad. We further find that the disparity in the reaction barrier can be attributed to the enzyme environment in the vicinity of the QM subsystem through an energy decomposition analysis. This finding helps researchers gain a deeper understanding of the catalytic mechanism of γ-secretase.

## 2 Methods

### 2.1 MD Simulations

All the simulations were performed using the AMBER18 package.^32^ The cryo-EM structure of γ-secretase bound with APP (PDB ID: 6IYC, shown in Figure 1A) served as the basis for conducting unbiased all-atom MD simulations.^30^ For this study, we selected only the transmembrane domain of γ-secretase, as the extracellular domain (residues 1–664) of NCT subunit was dispensable during the substrate recognition and cleavage.^22,33,34^ For APP, only the longest sequence resolved experimentally, namely, residues 37–55, was retained. The 6IYC structure contains D385A and Q112C mutations; therefore, these mutations were reverted. The N-termini and C-termini of all chains were capped with an acetyl group (ACE) and a methyl amide group (CT3), respectively.

The modified γ-secretase/APP complex structure was oriented using the Orientation of Proteins in Membrane (OPM) program^35^ and embedded in a phosphatidylcholine (POPC) lipid bilayer with the CHARMM-GUI online server.^36^ The system charges were neutralized with 150 mM NaCl. The CHARMM36m force field^37^ and TIP3P water model^38^ were employed for unbiased MD simulation. The system underwent a 5000-step energy minimization, followed by a six-step equilibration process. The first three equilibration simulations, totaling 375 ps, were performed at a constant volume and temperature (NVT ensemble) with a 1 fs time step. The subsequent three equilibration simulations, totaling 1.5 ns, were performed at constant pressure and temperature (NPT ensemble) with a 2 fs time step. During equilibration, positional constraints on the membrane and proteins were gradually released. Finally, multiple independent replicas of classical unbiased MD simulations were performed with *pme.CUDA* module of the AMBER18 program.^39^ In these production simulations, all bond lengths to hydrogen atoms were constrained with the SHAKE method.^40^ Langevin dynamics were used in all simulations with a collision frequency of 1.0 ps^–1^. Nonbonded and short-range electrostatic interactions were managed with a cutoff of 12 Å and an F-switch function of 10 Å. Long-range electrostatic interactions were described using the Particle Mesh Ewald summation method.^41^ The simulation temperature was set to 303.15 K and the pressure was set to 1 bar using a semi-isotropic coupling method. All unbiased MD simulation frames were saved every 100 ps.

### 2.2 QM/MM MD Simulations

The representative structures of active conformation in both protonation states depicted in Figures S2 and S3, selected as the center structure of the active conformational ensemble based on the root-mean-squared distance (RMSD) of the catalytic dyad and L49-V50 of the substrate, was utilized as the initial structure for QM/MM calculations. All QM/MM calculations were performed using the *sander.MPI* module within the AMBER18 program,^42^ with the QM region of the system encompassing L49, V50, D257, D385, and an activated water molecule. The QM region of the system was treated with the SCC-DFTB semiempirical method using the Slater-Koster mio-1-1 parameters.^43^ Four hydrogen “link atom”^44^ were used to model the bond across the QM/MM boundary. The total charge of the QM region was −1. The rest of protein and lipid molecules were described with the CHARMM36m force filed,^37^ and the rest water molecules were described by TIP3P force field.^38^ We used the QM/MM MD accelerated by enhanced sampling techniques to explore the formation process of gem-diol intermediates in APP catalyzed by the γ-secretase.

First of all, the reaction process was roughly explored using the metadynamics approach to generate the initial structures for subsequent umbrella sampling. The QM/MM metadynamics MD simulations were performed using the AMBER18 package patched with Plumed2 plugin,^45^ using two designed collective variables to describe the formation of the gem-diol intermediate. The details of additional parameters for metadynamics simulations are provided in Table S2. Repulsive walls were set for each of the distances, with a constant of 200 kcal/mol, to restrain the exploration to the reactive event. The sampled two CV values from metadynamics are plotted in Figure S5.

This chemical step was then modeled with the QM/MM umbrella sampling approach to obtain the full 2D-PMF along two collective variables (CVs). The first CV accounts for the proton transfer between the protonated catalytic residue and oxygen of carbonyl group of scissile peptide bond, defined as *CV*1 = *d*_O1–H_X__ – *d*_(O–H)_X__, where X is Asp257 or Asp385, ranging from −2.0 to 2.0 Å by a step of 0.1 Å. The second CV describes the nucleophilic attack and proton abstraction from the activated water, defined as *CV*2 = *d*_O_w_–H_w__ – *d*_O_w_–C1_, ranging from −2.0 to 1.5 Å by a step of 0.1 Å. The initial structure for each umbrella sampling window *i* obtained from the QM/MM metadynamics simulations is determined by a value *v*_*i*_ given by
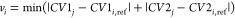
where *CV*1_*i*,ref_ and *CV*2_*i*,ref_ are the values of *i-*th window in umbrella sampling. *j* denotes the index of structures sampled from metadynamics simulations. *CV*1_*j*_ and *CV*2_*j*_ are the values of collective variables in the *j-*th structure. Therefore, the whole 2D-PMF consisted of a total of 1476 US windows, and the sampling time was 10 ps/window with a 1 fs time step. Two-dimensional potential of mean force (2D-PMF) profiles were generated using the weighted histogram analysis methods (WHAM)^46^ with a convergence criterion of 10^–5^. The minimum free energy path (MFEP) on the 2D-PMF was analyzed using the minimum energy path surface analysis (MEPSA) program.^47^ We divided the simulation data per umbrella sampling window into equal time intervals using blocks of 2 ps (0–2, 2–4, 4–6, 6–8, and 8–10 ps). The convergences of umbrella samplings in both protonated states were assessed by calculating 2D-PMF profiles at different time intervals (Figures S6–S10). It was observed that umbrella simulations could achieve convergence within 6 ps. Consequently, the 2D-PMF profiles based on a time region of 6–10 ps were selected for analysis in the main text.

### 2.3 Interaction Energy Decomposition

The interaction energy decomposition method was used to analyze how the active site residues stabilize or destabilize the TS in the gem-diol formation process of APP catalyzed by γ-secretase. This approach was first used by Bash et al. in the study of triosephosphate isomerase,^48^ and it has been extensively applied to a number of enzyme systems.^49−53^ The impact of an individual residue on the interaction energy of a given structure is estimated by examining the difference of energies when a specific residue is present (denoted by *i* in eq 1) or when it is substituted with Gly (denoted by *i* – 1 in eq 2) according to
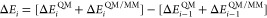
1where Δ*E*_*i*–1_^QM^ and Δ*E*_*i*–1_^QM/MM^ are the QM and QM/MM energies computed when the residue *i* has been replaced by Gly. The results are averaged. Then, from RS to TS, the differences between the stabilization effects for each residue are estimated by
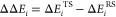
2

In the present analysis, the method was applied for residues within the 5 Å range of the QM region in the enzyme. This includes 32 residues for the active site of the enzyme–substrate complex. We used the structures saved during the umbrella sampling MD simulations, corresponding to the reactant state (RS) and transition state (TS) within a range of 0.1 Å of the reaction coordinate stationary points. For the protonation of Asp257, these two states are defined with the CV value within 0.1 Å, corresponding to *CV*1 = 0.6 Å/*CV*2 = −2.0 Å in RS and *CV*1 = −0.5 Å/*CV*2 = −0.7 Å. – 0.6 in TS, respectively. These states correspond to *CV*1 = 0.6 Å/*CV*2 = −2.0 Å in RS and *CV*1 = −0.4 Å/*CV*2 = −0.7 Å in TS for the other protonated system. To calculate the average values for the interaction energy and stabilization effects (⟨ΔΔ*E*_*i*_⟩) of each residue, a total of 150 snapshots from umbrella sampling MD simulations were considered, with reaction coordinates corresponding to RS and TS.

### 2.4 Analysis

Post-processing analysis was done using the CPPTRAJ tool.^54^ Visual molecular dynamics (VMD) was used to manipulate the structures and generate images for visual inspection.^55^ The calculations of distances, angles, and the selection of active conformations were conducted with the *MDAnalysis* module for Python.^56^

## 3 Results and Discussion

### 3.1 Different Conformational Properties of γ-Secretase in Different Protonation States of Catalytic Dyad

We performed multiple replicas of unbiased MD simulations for each protonation state of the catalytic dyad (Table 1). The accumulated simulation time for each protonation state reached over 100 μs. Following previous studies,^19,20,57,58^ we characterized the conformational states of γ-secretase by examining the conformational free energy Δ*G*_dyad_ as a function of the distance between the two catalytic aspartic acid residues (*dd*_Asp_), calculated according to
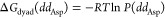
3

**Table 1:** Summary of Unbiased MD Simulations in Different Protonation States^a^

simulation system	*Nr*	*Ns*	*T* (K)	simulation length (μs)	type	*Ns*_a_
D257-P	40	1 054 355	303.15	105.4	Asp257i_385i	180
					Asp257i_385o	17
					Asp257no_385o	977
					Asp257no_385i	1523
D385-P	40	1 015 778	303.15	101.6	Asp385i_257i	245
					Asp385i_257o	49
					Asp385no_257o	5113
					Asp385no_257i	263

a *Nr* denotes the number of simulation replicas. *Ns* denotes the number of total sampled structures in all accumulated simulations. *Ns*_a_ denotes the number of sampled active structures in the different protonation states.

**Figure 2:**
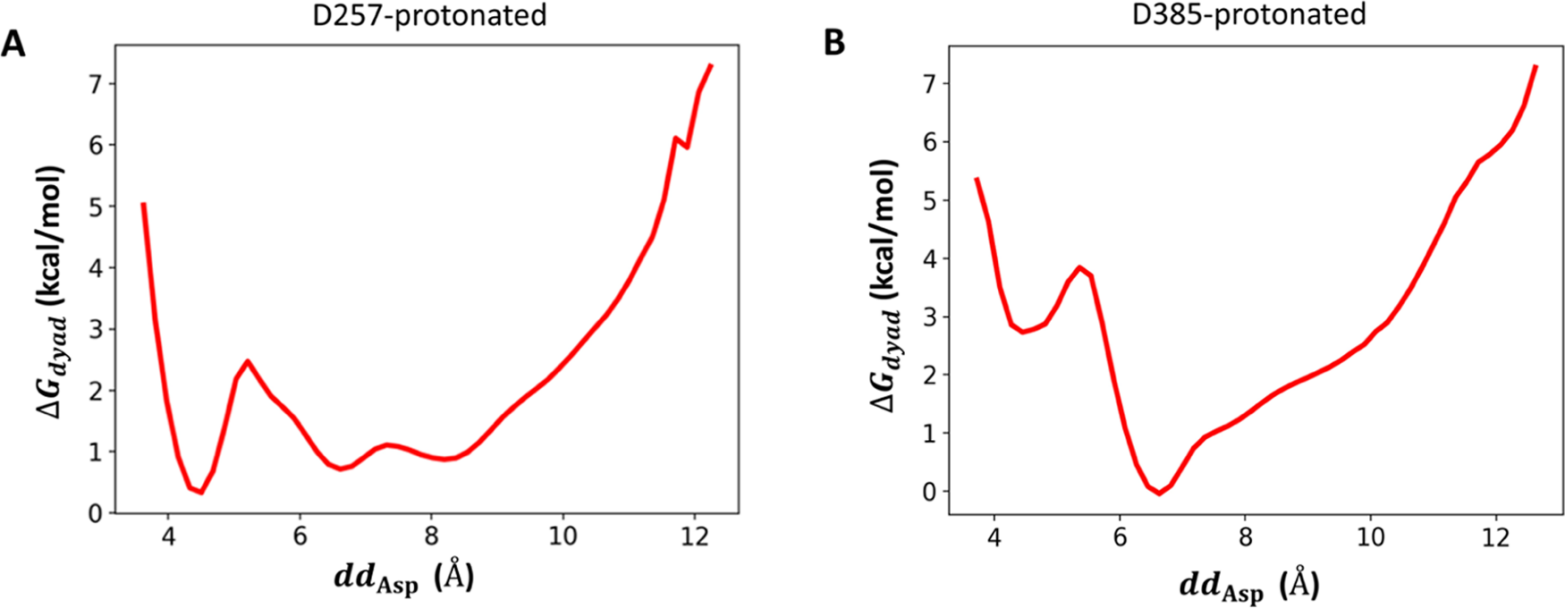
Free energy profiles Δ*G*_dyad_ along the *dd*_Asp_ in the extensive unbiased simulations with the D257-protonated state (A) and D385-protonated state (B). The Δ*G*_dyad_ is defined as −*RT* ln *P*(*dd*_Asp_). The *dd*_Asp_ distance is measured as the distance of the Cγ atom of D257 and the Cγ atom of D385.

Figure 2 shows Δ*G*_dyad_ (*dd*_Asp_) when either Asp257 or Asp385 is protonated. The results reveal distinct free energy minima in the different protonated states. There are three minima at *dd*_Asp_ = ∼4.5, ∼7, and ∼8.5 Å when Asp257 is protonated (Figure 2A). The first minimum at ∼4.5 Å corresponds to the formation of a direct HB between Asp257 and Asp385. A second one at ∼7 Å is characterized by a water molecule that bridges the two aspartic acids. For a third one at ∼8.5 Å, the two aspartic acids lost the bridging water molecule and departed farther from each other. The first minimum is slightly more favorable than the other two, and there is only a small barrier of about 2 kcal/mol separating the first minimum for the others. In contrast, when Asp385 is protonated, only a major conformational state at ∼7 Å, corresponding to the water-bridged structures, is observed (Figure 2B). The free energy of the minimum at ∼4.5 Å is increased significantly, and the enzyme needs to overcome a barrier of ∼4 kcal/mol to access this minimum. Taken together, these results suggest that the catalytic dyad is more flexible in the D257-protonated state than in the D385-protonated state.

Previous coarse-grained (50 replicas of 1 μs) MD simulations based on the apo structures sampled the structures with *dd*_Asp_ < 5 Å in the D385-protonated state.^58^ Based on the holo-state structures, the average distance of *dd*_Asp_ at ∼6.4 Å is observed through unbiased atomistic MD simulations (3 replicas of 1 μs) in both protonated states.^57^ However, through GAMD simulations,^19,20^*dd*_Asp_ are found distributed between 4 and 10 Å.^19,20^ Thus, the values for *dd*_Asp_ observed in our long unbiased simulations are largely in good agreement with the observations from the accelerated simulations.

**Figure 3:**
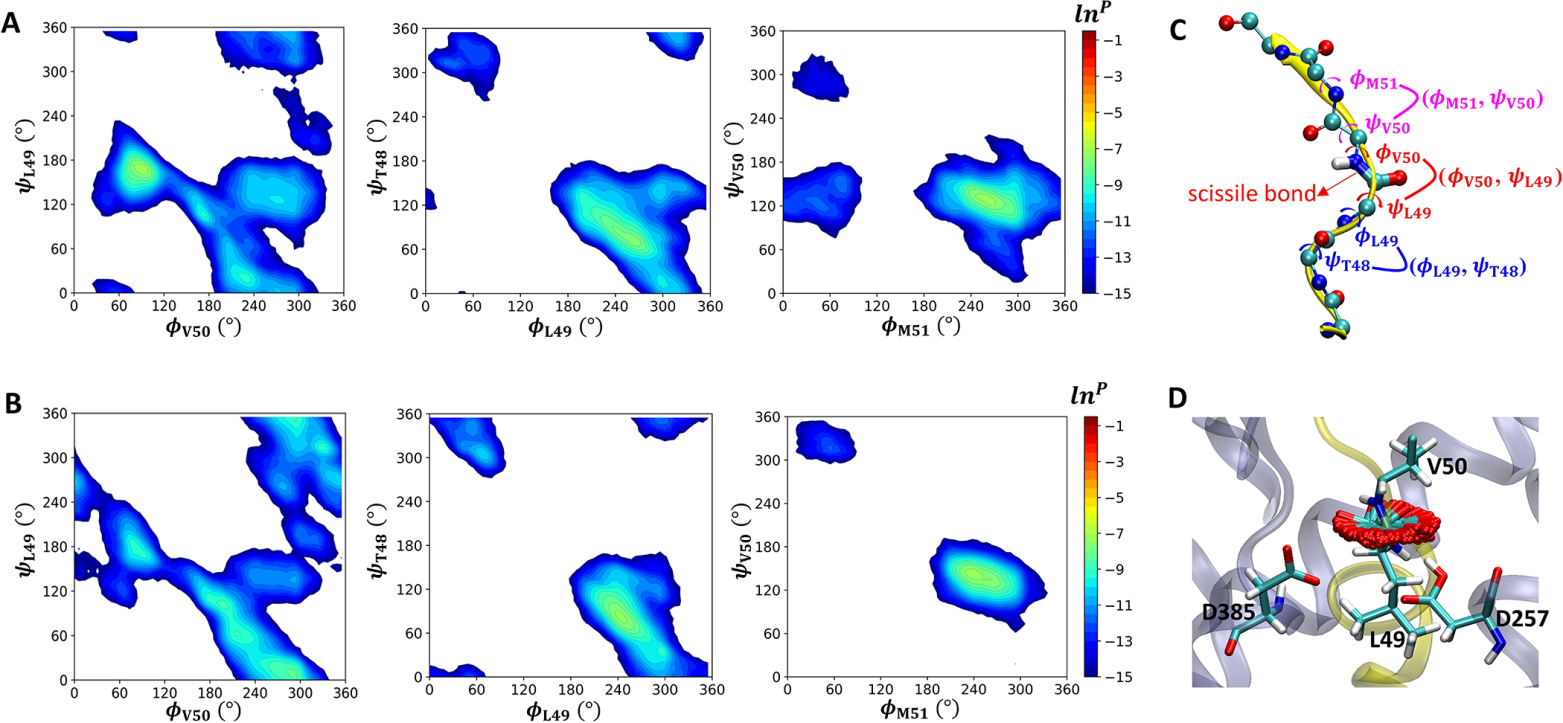
Backbone dihedrals near the scissile peptide bond in the extensive unbiased simulations with the D257-protonated state (A) and the D385-protonated state (B). Left of (A, B): logarithm of probability distributions of (ϕ_V50_, ψ_L49_) which are adjacent to the scissile peptide bond; Middle of (A, B): logarithm of probability distributions (ϕ_L49_, ψ_T48_); Right of (A, B): logarithm of probability distributions of (ϕ_M51_, ψ_V50_). (C) Positions of (ϕ_V50_, ψ_L49_), (ϕ_L49_, ψ_T48_), and (ϕ_M51_, ψ_V50_) in the substrate. (D) Rotation of the carbonyl group of the scissile peptide bond in the protonation of Asp257.

Apart from the catalytic dyad, we further examined the conformational dynamics of the bound substrate, especially in the nearby region where the scissile peptide bond is located. As the orientation of a peptide bond is determined by the torsions of its adjacent backbone bonds, the rotation of the scissile peptide bond (L49-V50) can be characterized by the dihedral pair (ϕ_V50_, ψ_L49_). As shown in Figure 3, the probability map of this dihedral pair displays a wide distribution across multiple distinct regions when either Asp257 or Asp385 is protonated, indicating that the scissile bond is highly flexible for both protonation states. There is a difference in the preferred orientations of the scissile bond between the two protonation states. This is mainly because the scissile bond needs to point its dipole in different manners in response to distinct local electrostatic environments created by the catalytic dyad in different protonation states. On the contrary, the peptide bonds adjacent to the scissile bond rotated much less, as indicated by the narrow distributions of the corresponding torsion pairs, largely concentrated within single well-defined regions (Figure 3). Taken together, these results suggest that the bound substrate has rather conserved internal conformations, except for its scissile bond.

### 3.2 Active Conformations of γ-Secretase in Different Protonation States of Catalytic Dyad

**Figure 4:**
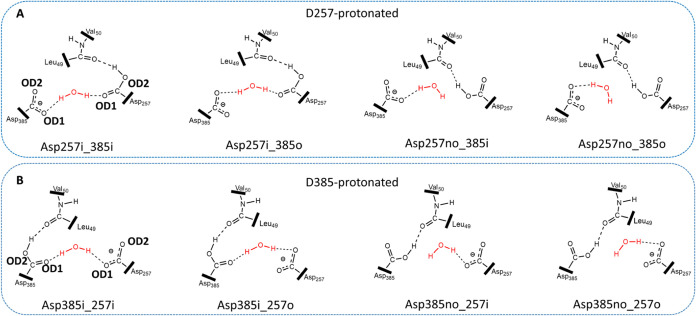
Possible four protonation states of the catalytic Asp dyad (i = inner oxygen (OD1) and o = outer oxygen (OD2)) for hydrolysis with the D257-protonated state (A) and D385-protonated state (B). In each protonated system, the bridged lytic water was hydrogen-bonded to both catalytic dyads in the first two types of protonation states, and the lytic water was only hydrogen-bonded to the deprotonated aspartate in the latter two types of protonation states.

Next, we sought to examine if the active state of γ-secretase could be accessed in our unbiased simulations starting from the experimentally revolved inactive conformation. Different active site conformations in the active state in protease could be adopted based on previous studies.^10,19,20,59,60^ As a slight reorientation in the position of the aspartate residues can make another set more favorable for catalysis. By considering the two possible orientations of each carboxylate group, we collected four sets of protonation states for each protonated system, henceforth referred to as Asp257i_385i, Asp257i_385o, Asp257no_385o, Asp257no_385i in the protonation of Asp257 and Asp385i_257i, Asp385i_257o, Asp385no_257o, and Asp385no_257i in the protonation of Asp385 during the unbiased MD simulations (Figure 4 and Table 1). For either protonated system, the first two types of protonation states, the catalytic dyad is bridged with the lytic water through two hydrogen bonds, but the lytic water has only one hydrogen bond with deprotonated aspartate in the latter two types.

**Figure 5:**
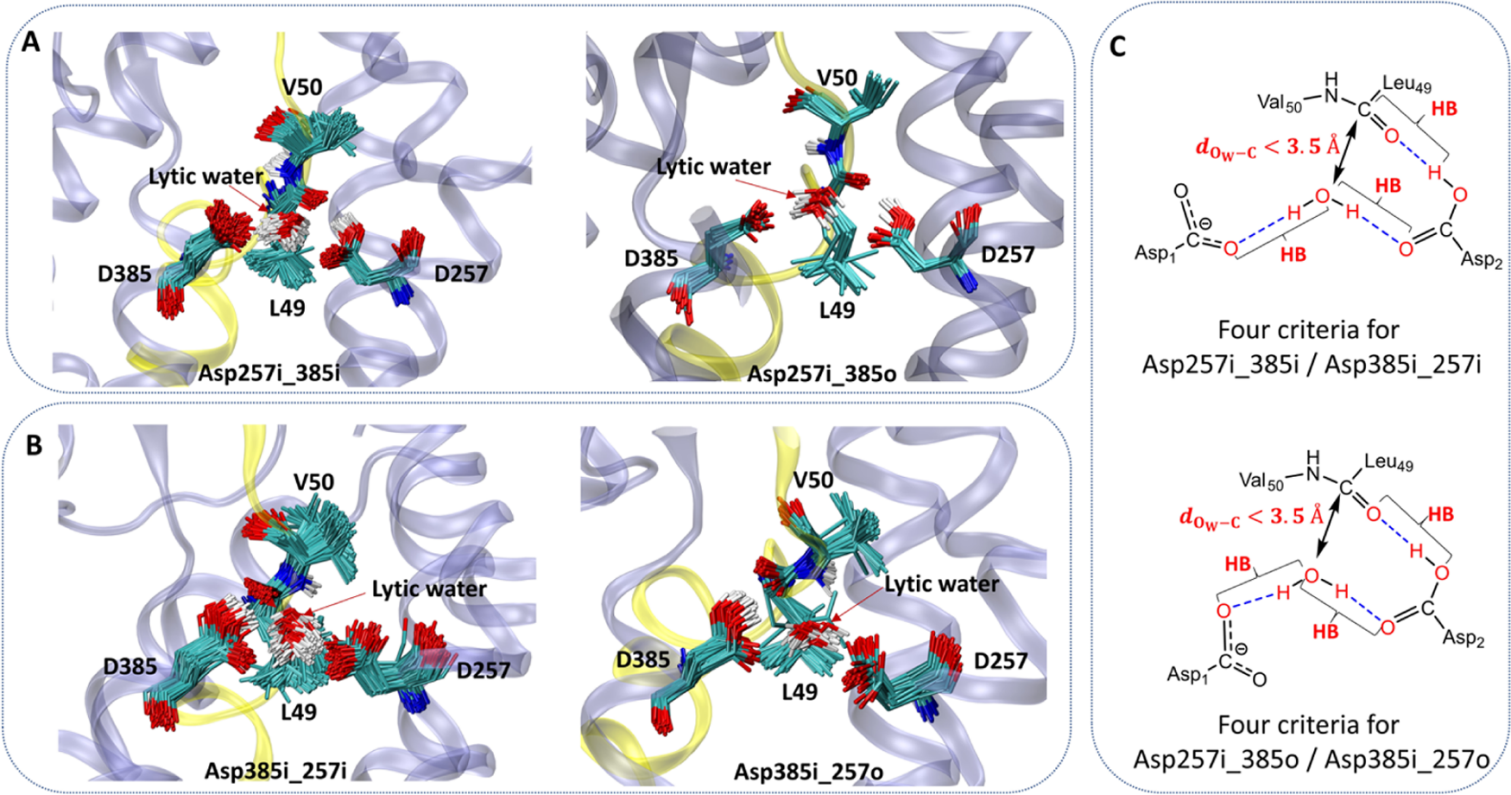
Superimposed active structures of the first two types in the D257-protonated system (A) and the D385-protonated system (B). (C) Four criteria to determine the first type (top) and the second type (bottom) in both protonation states.

We employed several criteria proposed by other groups^19,59,60^ to detect the occurrence of different types of protonation states for each protonate system in our simulations (Figures 5 and S1). These criteria for the first two types include: (1) the formation of a hydrogen bond (HB) between the protonated Asp of the catalytic dyad and the carbonyl oxygen atom of the scissile bond; (2) the presence of lytic water in close proximity of the carbonyl carbon atom of the scissile amide bond with *d*_O_w_–C_ < 3.5 Å; (3) an HB formed between the deprotonated Asp of the dyad and the lytic water hydrogen for water activation; (4) an additional HB formed between the other lytic water hydrogen and the protonated Asp that has been proposed to further facilitate the reaction. Based on these criteria, our analysis has identified numerous active conformations for both protonation states of the catalytic dyad (Figure 5). The probabilities of finding the active conformations for the first two types are similar between the two protonation states (0.0002 versus 0.0003), indicating that the active state is equally accessible when either catalytic aspartate is protonated. The probabilities of finding the active conformations for the latter two types in both protonated systems (0.0023 versus 0.0053) are approximately 10–18 times those in the first two types of protonation states. This difference in state probabilities between the first two types and the latter two types in both protonated systems is easily understood, as this scenario in the active center only requires the formation of a single hydrogen bond. In the two latter types of protonation states for two protonated systems, the frequent states are Asp257no_385i and Asp385no_257o, respectively. Through QM/MM MD method, we first explored the reaction process of gem-diol intermediate in which the reactant was started from the most frequent states for each protonated system, namely Asp257no_385i (the highest occurrence, ∼0.0014) for the protonation of Asp257 and Asp385no_257o (the highest occurrence, ∼0.0050) for the protonation of Asp385 (Figures S8, S9, S11, and S12). The reaction energy barriers calculated were very high (>40 kcal/mol) for the pathways starting from these two types of protonation states (Figures S11 and S12). Note here we assume the less frequent protonation states in the latter two protonation states, namely Asp257no_385o and Asp385no_257i, adopt a reaction mechanism similar to that we have already calculated in Asp257no_385i and Asp385no_257o. Thus, the latter two types of protonation states for either protonated system disfavor the reaction of gem-diol formation. Among the first two types of protonation states, Asp257i_385i and Asp385i_257i have been reported by previous work of γ-secretase,^19,20,61^ whereas Asp257i_385o and Asp385i_257o have not yet been reported by researchers. A lytic water could be recruited through two hydrogen bonds with a catalytic dyad of enzyme in these types which are well poised for ε cleavage of the amide bond between L49-V50 of APP. Therefore, we focus on the first two types of protonation states in both protonated systems to investigate the mechanistic detail regarding the formation of gem-diol intermediates in the present study.

**Figure 6:**
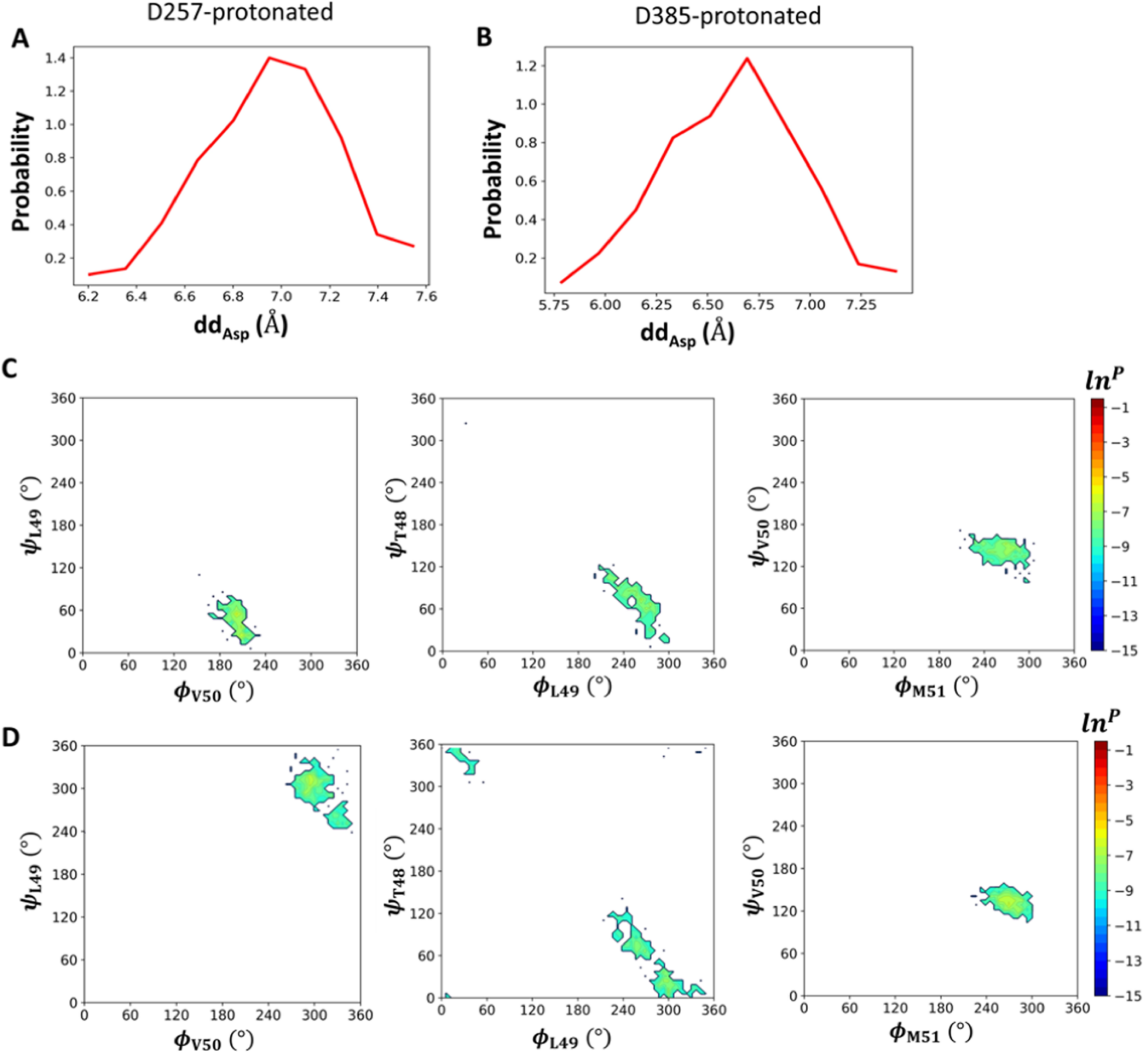
Distribution of *dd*_Asp_ and backbone dihedrals near the scissile peptide bond in the first two types of active conformations with the D257-protonated state (A, C) and the D385-protonated state (B, D). Left of (C, D): logarithm of probability distributions of (ϕ_V50_, ψ_L49_) which are adjacent to the scissile peptide bond; middle of (C, D): logarithm of probability distributions (ϕ_L49_, ψ_T48_); right of (C, D): logarithm of probability distributions of (ϕ_M51_, ψ_V50_).

We further probed the conformational features of the catalytic dyad in the first two types when the enzyme is in its active state. Again, the conformation of the catalytic dyad is probed with *dd*_Asp_. As shown in Figure 6, in the D257-protonated state, *dd*_Asp_ is narrowly distributed between 6.2 and 7.4 Å and centered at ∼7 Å. In the D385-protonated state, a narrow distribution of *dd*_Asp_ between 6.0 and 7.2 Å is observed. This distribution peaked at ∼6.75 Å, slightly shorter than the preferred distance between the catalytic aspartates in the D257-protonated state.

The conformations of the bound substrate in the active state are also examined. We analyzed the backbone torsions of the key substrate part harboring the scissile bond (L49-V50) and other nearby peptide bonds (T48-L49 and V50-M51). Figure 6 shows the probability distributions of the torsion pairs associated with these peptide bonds. For T48-L49 and V50-M51, their corresponding torsion pairs, namely (ϕ_L49_, ψ_T48_) and (ϕ_M51_, ψ_V50_), are distributed in the same regions regardless of whether the enzyme is in the active state (Figures 3 and 6), indicating that these peptide bonds adjacent to the scissile bond do not need to change their conformation in the active state. On the other hand, in the active state, the torsion pair (ϕ_V50_, ψ_L49_) for the scissile bond is found narrowly distributed in only one of many regions that are generally accessible for this torsion pair (Figures 3 and 6). Taken together, these results suggest that the enzyme needs to reorient only the scissile bond of the bound substrate in specific directions to access the active conformation, and such a minute conformational change needs to hinge only on the ability of the scissile bond to rotate.

### 3.3 γ-Secretase in Different Protonation States of Catalytic Dyad Exhibits Distinct Reaction Barriers of Gem-Diol Formation

Having obtained the active conformations, we proceed to investigate the efficiency of γ-secretase in catalyzing the formation of gem-diol, as this step has been found by numerous previous studies to limit the overall hydrolysis rate.^9,10,14^ The reactions under both protonation states are considered. For either protonation state of the dyad, a structure selected as the center of the active conformational ensemble was used to build a hybrid QM/MM model of the enzyme (see the Section 2). The catalytic dyad, the scissile bond, and the residue adjacent residues to this bond, and the lytic water are included in the QM region of the model. The potential energy of this QM part is described with the self-consistent charge density functional tight binding (SCC-DFTB) method using the Slater-Koster mio-1-1 parameters, which is an approximate density functional theory (DFT)-based method.^43^ This method has been extensively used for biological systems and provides a relatively good trade-off between the speed of calculation and the accuracy as compared to *ab* initio approaches.^62−66^ The application of the SCC-DFTB method in the elucidation of enzymatic hydrolysis reactivity involving aspartic residue has been reported by several previous studies.^67,68^ The rest parts of the system are represented again with the CHARMM36m all-atom force field.^37^

To model the formation of gem-diol intermediates in both protonated systems, we have designed two collective variables (CVs) to monitor the reaction progress (Table S2): one CV (*CV*1) describes the proton transport from the protonated aspartic acid of the dyad to the carbonyl oxygen of the scissile bond and the other (*CV*2) describes the nucleophilic attack of the lytic water to the carbonyl carbon. To obtain reaction paths in an unbiased manner, we first construct the two-dimensional potential of mean force (2D-PMF) as a function of both CVs. To this end, we have computed the 2D-PMFs with a two-step procedure detailed in the Method. This procedure first explores the reaction landscape with a metadynamics technique using the two CVs. The conformations generated from the metadynamics simulations are then used to launch a 2D grid of umbrella sampling simulations with the same CVs to get an accurate and converged estimate of the PMFs (Figures S6 and S7).

**Figure 7:**
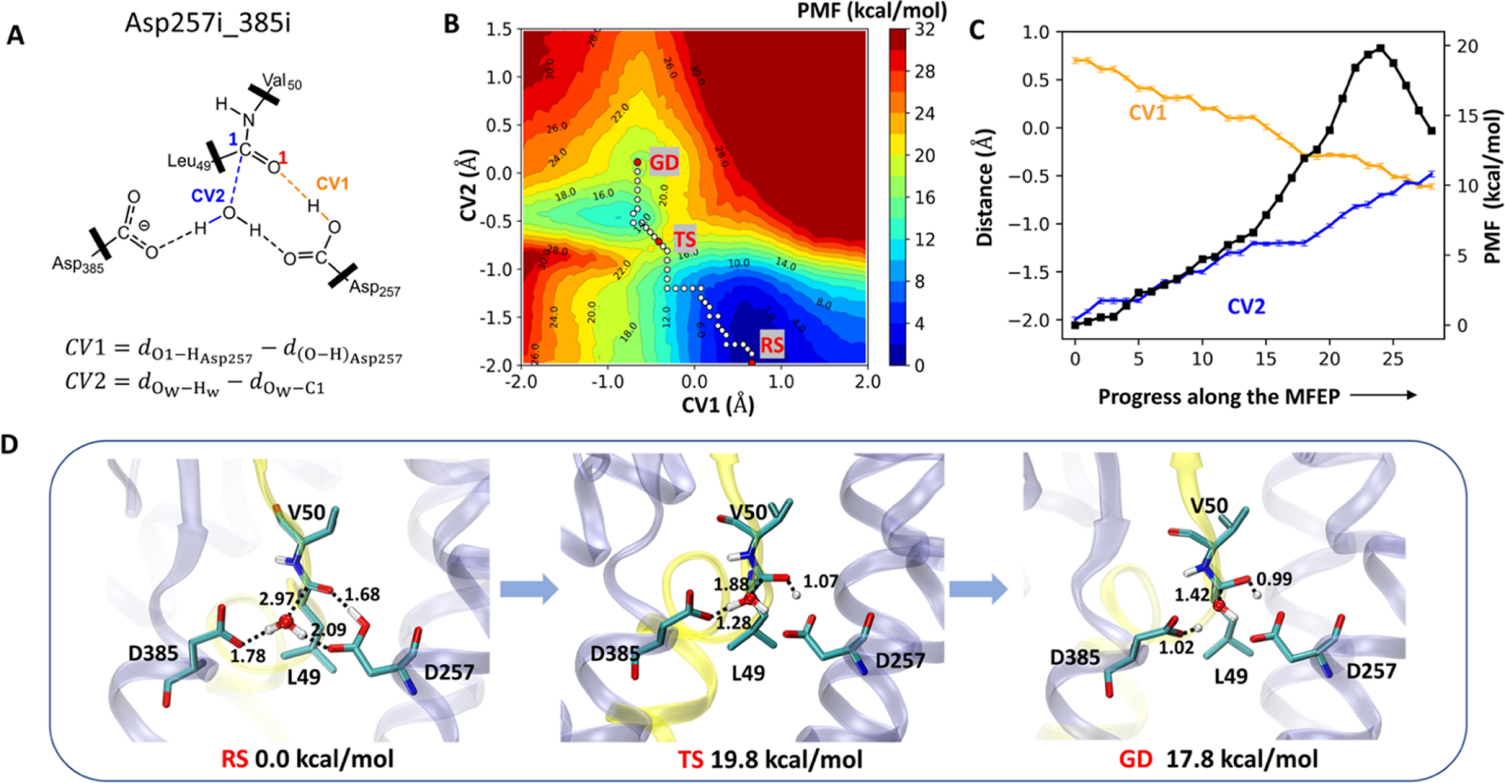
Summary of the formation of gem-diol catalyzed by γ-secretase calculated at the SCC-DFTB/CHARMM36m level with the D257-protonated system. The reactant state was from Asp257i_385i of active site conformation. (A) Two CVs were used to conduct the umbrella sampling. (B) The reconstructed reaction 2D-PMF along defined two CVs. The white and red circles show the free energy profiles along the MFEP. RS, TS, and GD denote the reactant state, transition state, and gem-diol intermediate state, respectively. (C) Values for the reaction free energy profile (black line), CV1 (orange line), and CV2 (blue line) along the MFEP. The error bars are marked in every point along the MFEP. (D) Structures of the key states in the gem-diol formation catalyzed by γ-secretase. The labeled distances are given in Å. The two CVs values for three structures: *CV*1 = 0.6 Å/*CV*2 = −2.0 Å in RS, *CV*1 = −0.4 Å/*CV*2 = −0.7 Å in TS, and *CV*1 = −0.7 Å/*CV*2 = 0.1 Å in GD.

**Figure 8:**
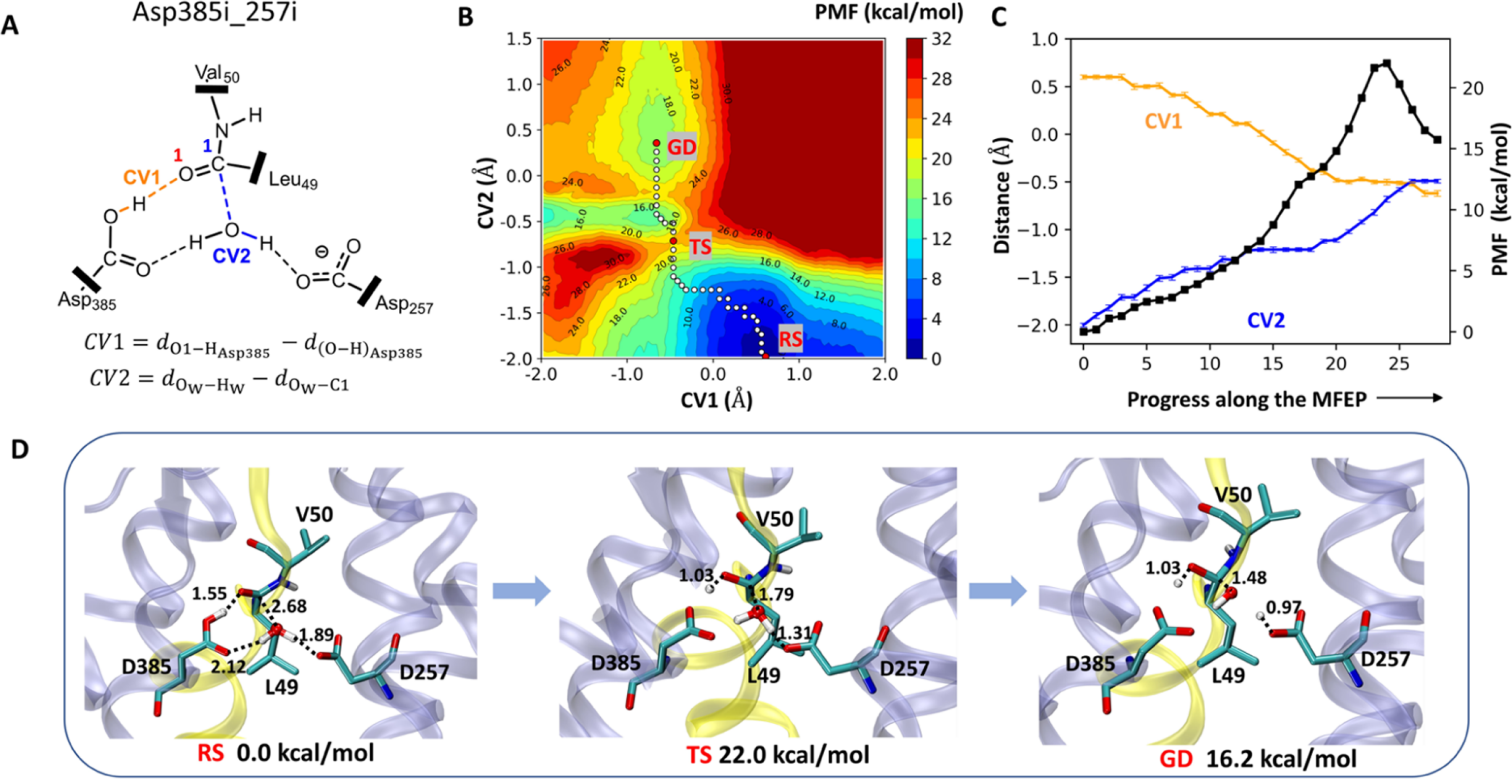
Summary of the formation of gem-diol catalyzed by γ-secretase calculated at the SCC-DFTB/CHARMM36m level with the D385-protonated system. The reactant state was from Asp385i_257i of active site conformation. (A) Two CVs were used to conduct the umbrella sampling. (B) The reconstructed reaction 2D-PMF along defined two CVs. The white and red circles show the free energy profile along the MFEP. RS, TS, and GD denote the reactant state, transition state, and gem-diol intermediate state, respectively. (C) Values for the reaction free energy profile (black line), CV1 (orange line), and CV2 (blue line) along the MFEP. The error bars are marked in every point along the MFEP. (D) Structures of the key states in the gem-diol formation catalyzed by γ-secretase. The labeled distances are in Å. The two CVs values for three structures: *CV*1 = 0.6 Å/*CV*2 = −2.0 Å in RS, *CV*1 = −0.5 Å/*CV*2 = −0.7 Å in TS and *CV*1 = −0.7 Å/*CV*2 = 0.4 Å in GD.

Figures 7B and 8B show the 2D-PMF landscapes obtained under both protonation conditions of the catalytic dyad. According to the analysis of the minimum free energy path (MFEP) on both 2D-PMFs, there is no intermediate before the transition state (TS) for the formation of the gem-diol intermediate, which implies a concerted mechanism. The calculated free energy barriers with respect to the reactant state (at the SCC-DFTB/CHARMM36m level) for the generation of the gem-diol intermediate in both protonation states are 19.8 and 22.0 kcal/mol, respectively. As shown in Figure S13, the calculated free energy barrier for the pathway starting from the Asp385i_257o conformation with respect to the reactant state (at SCC-DFTB/CHARMM36m level) is 23.7 kcal/mol which is ∼4 kcal/mol higher in the protonation of Asp257. Previous QM calculation at the B3LYP level that considered all atomic details of a small region encompassing the catalytic site reported that the barriers for this step varied between 16.6 and 24.4 kcal/mol for different scissile bonds when Asp257 was protonated.^10^ In addition, the barriers for the formation of gem-diol intermediates are also estimated for the other soluble aspartyl proteases, such as renin and HIV-1 protease, using the QM/MM method with QM accuracy at an M06 or BLYP level. The barriers for this step are calculated to be 22.0^9^ and 18.0 kcal/mol^69^ for these two enzymes, respectively. Thus, the energy barrier values in our results are in line with the previously reported values.

Of note, although we observed the expected gem-diol intermediate at (*CV*1 = −0.7 Å, *CV*2 = 0.1 Å) in which the lytic water gave one of its proton to the initially deprotonated Asp after the transition state (TS) for the gem-diol formation,^9,10,17,70^ we also observed another gem-diol intermediate in which the lytic water gave its proton to the initially protonated rather than deprotonated Asp in both protonation states (Figure S14). As Figure S14 shows, the same values for the collective variables are between the two protonation states (*CV*1 = −0.7 Å, *CV*2 = −0.5 Å). We further examined the stability of this unexpected intermediate by conducting QM calculation at the B3LYP-D3/6-31G(d,p) and SCC-DFTB level in both protonated states (Figure S18 and Table S4). The structures in the starting point of optimization were from the unexpected gem-diol (GD’) intermediates extracted from the QM/MM system using both SCC-DFTB and B3LYP methods. The expected gem-diol (GD) intermediates were found at the end point of optimization for both protonation states in both SCC-DFTB and B3LYP-D3 methods. The expected gem-diol (GD) intermediates were validated by visual inspection and normal model calculation (no imaginary frequency in either method for both protonation stats). As Table S4 shows, the relative energy values varied with the level of accuracy of the QM methods employed, but the relative energies of GD intermediates are at least 4 kcal/mol lower than that in GD’ intermediates calculated at the same accuracy level. The result shows that this unexpected intermediate appeared to be nonmetastable using both SCC-DFTB and B3LYP-D3 for both protonation states, indicating that there might be the relative stability of different gem-diol intermediates during the catalytic process in γ-secretase caused by enzyme environment. Nevertheless, our study is focused on the barriers of the gem-diol formation, which takes place before these intermediates along the reaction path. As shown in Figure S17, the reaction path identified with our QM/MM calculation agrees with the one derived from the QM calculation from the reactant to the TS in both protonated states. The estimated barriers are also similar between the two methods in both protonated states (Figure S16). Moreover, we are mainly concerned with the barrier difference between the reaction paths for different protonation states of the catalytic dyad. The discrepancy discussed above may cancel out. Thus, the conclusion from this study should not be affected qualitatively due to the use of the SCC-DFTB method.

A key prediction from the QM/MM calculation is that the barriers for the gem-diol formation could be ∼2.2 kcal/mol lower in the Asp257-protonated state than in the Asp385-protonated state. Thus, the protonation of Asp257 could bring about more reaction efficiency to the enzyme than the protonation of Asp385. In the previous studies^18,20,21,57^ that determined favorable protonation states based on p*K*_a_ calculation, the p*K*_a_ difference between the favorable and unfavorable aspartates for protonation are mostly estimated to be 0.8–3.7 p*K*_a_ unit, corresponding to a free energy difference of about 1.2–5.5 kcal/mol. If the difference in reaction barriers between the two protonation states is considered, then the previous conclusions in favor of the protonation of Asp385 based only on the p*K*_a_ calculation would be significantly weakened or even reversed.

### 3.4 Rationalization of Difference in Reaction Barriers

It would be helpful to find the possible cause of the difference in the reaction barriers between the two protonation states of the catalytic dyad.

We have conducted DFT calculations on a small gas-phase model extracted directly from the QM/MM geometry in both protonation states (Supporting Information section “Benchmarking”). The SCC-DFTB method was used to compute the energetics of the QM subsystem in the QM/MM system described above in both protonation states. Here, we compare SCC-DFTB to more accurate methods on the reduced system to determine whether it gives reasonable results for two protonation states. The SCC-DFTB method was compared to B3LYP-D3 functional using both the 6-31G(d,p) and def2-TZVP basis sets by calculating the Δ*G* of the formation reaction of the gem-diol. It is important to note that TS could not be optimized in the gas phase with the SCC-DFTB method, as no saddle point was indicated between the RS minimum and the gem-diol tetrahedral intermediate. A similar challenge was encountered by Hirvonen et al. in their study of another hydrolase system, particularly among the OXA-48-like enzymes. They observed that despite the underestimation of barriers calculations of single-point energies using SCC-DFTB or more accurate QM methods consistently revealed the same potential energy surface shape. This finding suggests that the underestimation of energy barriers with SCC-DFTB does not affect the trends in reaction barriers.^65^ We calculated the energy difference from single-point energies at the SCC-DFTB and B3LYP-D3 functional with different basis sets between the B3LYP-D3/6-31G(d,p) optimized RS and TS structures. As Figure S16 shows, although the calculated barriers varied with the level of accuracy of the QM methods employed, the QM calculations at the same accuracy level revealed little difference (<0.5 kcal/mol, Table S3) in the energy barriers for the two protonation states of the catalytic dyad. In contrast, there is a non-negligible barrier discrepancy (∼2.2 kcal/mol) observed from the QM/MM calculation. Thus, this result suggests that the difference in the computed reaction barrier between the two protonation states should largely arise from the MM part of the enzyme.

**Figure 9:**
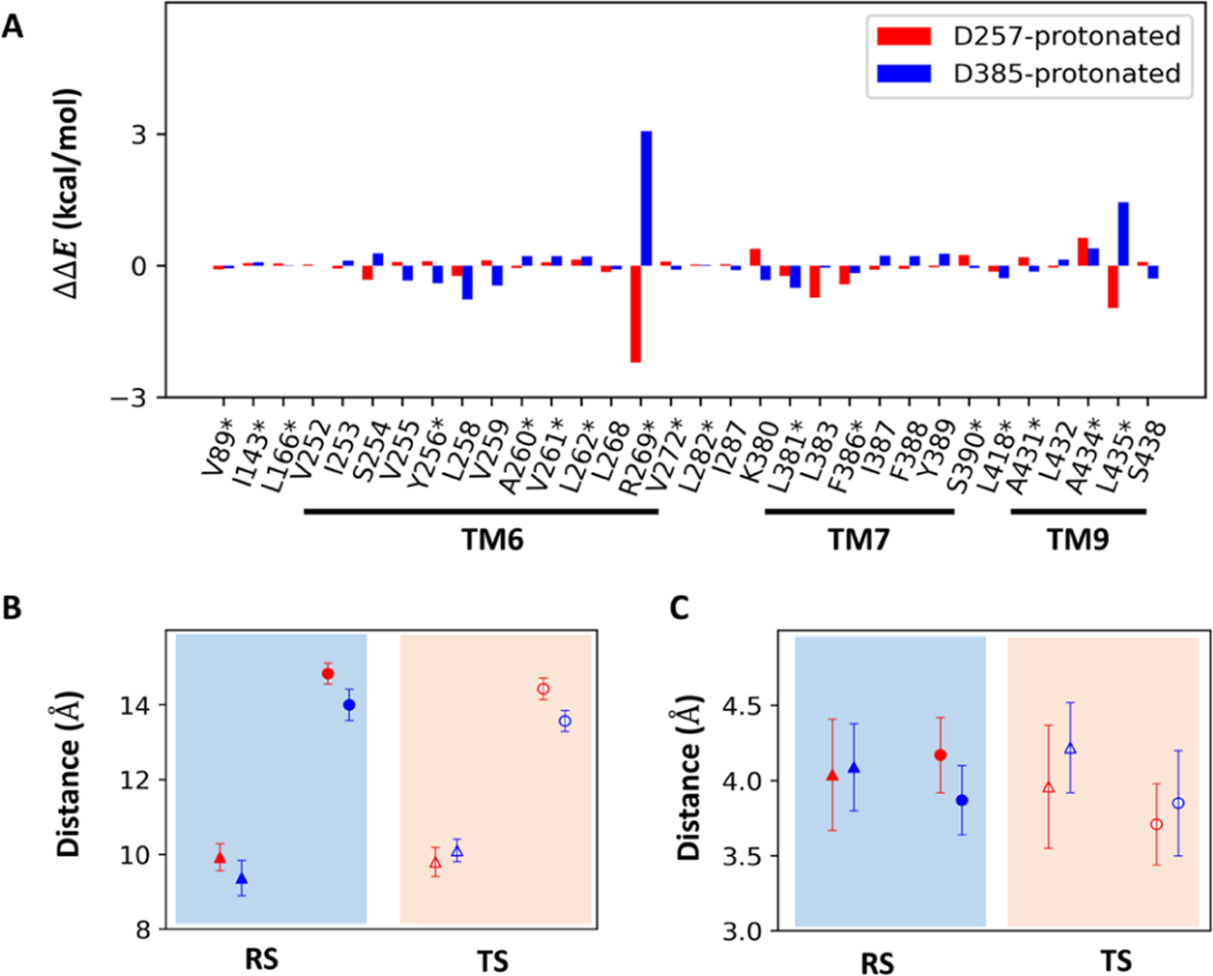
(A) Per residue in the vicinity of the QM subsystem contribution to stabilize or destabilize the transition state with respect to the reactant. Asterisks denote the mutations which have been studied experimentally.^8^ (B) Mean distance between Arg269 (center of mass of charged nitrogen atoms) and Asp257 (the closest side-chain oxygen atom) or Asp385 (the closest side-chain oxygen atom). The red triangles and red circles indicate the distances between Arg269 and Asp257 or Asp385 in the D257-protonated system, respectively. The blue triangles and blue circles denote the distance between Arg269 and Asp257 or Asp385 in the D385-protonated system, respectively. (C) Mean distance between Leu435 (the closest side-chain carbon atom) and the Asp257 (the closest side-chain oxygen atom) or Asp385 (the closest side-chain oxygen atom). The red triangles and red circles indicate the distance between Leu435 and Asp257 or Asp385 in the D257-protonated system, respectively. Blue triangles and blue circles denote the distance between Leu435 and Asp257 or Asp385 in the D385-protonated system, respectively. (B, C) For both protonated systems, the solid and hollow symbols show the RS and TS, respectively. The error bars are marked at every point.

To further understand the observed difference, we performed interaction energy decomposition analysis for the RS and TS in both protonation states. At first glance, Figure 9A shows the influence of individual residues that surround the active site on the reaction barriers, estimated as the change in the barrier height ΔΔ*E* if a residue would be mutated into a glycine (see the Section 2). A negative value indicates that the residue plays a role in lowering the barrier. Overall, the residues within 5 Å of the QM region altogether contribute favorably to the reaction barrier by −3.5 kcal/mol for the D257-protonated state but unfavorably by 2.7 kcal/mol for the D385-protonated state. Of note, the reaction barrier was predicted to be ∼7.2 kcal/mol higher if D385 is protonated. This difference agrees qualitatively with the barrier difference (∼2.2 kcal/mol) from our QM/MM free energy calculations, although it is greater in magnitude, probably because our analysis was based on energy instead of free energy. Thus, our result suggests that through direct interactions with the reaction center, the enzymatic environment is more capable of reducing the reaction barriers when D257 rather than D385 is protonated.

As Figure 9A shows, the disparity in energy barriers is a collective effect, with a larger number of residues stabilizing the TS in the protonation of Asp257. These residues analyzed in our study are primarily located in the TM6, TM7, and TM9 of the catalytic PS1 subunit, with 17 of these residues (denoted by asterisks in Figure 9A) having been previously subjected to experimental pathogenic mutagenesis studies.^8^ An interesting observation is that some residues seem able to stabilize the transition state more than the others in the protonation of Asp257 but destabilize the transition in the other protonation state. For instance, Arg269 exhibits opposite trends in the impact on transition state in two protonation states. Arg269 is one of two basic residues surrounding the active site. Furthermore, regardless of the protonation state, the basic side chain of Arg269 remained relatively close to the side chain of Asp257 (<10 Å) but kept further away from that of Asp385 (∼15 Å) during the reaction (Figure 9B). When Asp257 is protonated, the side chain of Asp257 undergoes a shift from a neutral form in RS to a state with a more negatively charged character in TS (Figure 7D). The presence of the nearby basic side chain of Arg269 can thus stabilize this shift of charge character of Asp257. In contrast, when D385 is protonated, the charge character of Asp257 changes from a negatively charged form in RS to a neutral form in TS. Thus, the presence of Arg269 nearby Asp257 disfavors the reaction by stabilizing the RS. Intriguingly, the important role of Arg269 in the chemical reaction is consistent with mutagenesis studies in which the replacement of this residue to glycine results in a decrease, but not a complete elimination, of its catalytic activity.^8^ In addition, Leu435 is another residue that has opposite impacts on the reaction barriers for the two protonation states although the impacts are less profound. Our analysis revealed that this residue forms intricate van der Waals contacts (<4.5 Å at the RS and TS in both protonated systems) of the side chains of the catalytic dyad (Figure 9C). This result corroborates the experimental finding that the replacement of Leu435 with a phenylalanine has resulted in a significant reduction in catalytic activity.^8^ Taken together, the specific residues identified here could be crucial sites for targeted mutations to reduce the reaction barrier during this chemical step and boost the enzyme’s efficiency or selectivity within the catalytic process.

## 4 Conclusions

The cleavage of APP by γ-secretase is a central molecular event in the production of disease-linked Aβ proteins. The working mechanism of this enzyme involves multiple physical and chemical processes, most of which remain largely elusive. In this study, we employed a multiscale computational approach to investigate the chemical reaction responsible for the actual cleavage of the peptide bonds at the ε position of APP. Through extensive unbiased all-atom simulations of the enzyme–substrate complex, we find that the enzyme can access the conformational ensemble in which the two aspartates of the catalytic dyad, namely, Asp257 and Asp385, are separated by varying distances and the scissile bond is oriented differently. Among these conformations, the active conformation that is ready for the reaction is accessible through the conformational fluctuation of the enzyme on a time scale of tens of microseconds. Our QM/MM simulations further show that although the commonly reported concerted formation of a gem-diol intermediate also occurs for this enzyme, the reaction efficiency of this step is found to be sensitive to which of aspartates in the catalytic dyad is protonated. The reaction barrier is predicted to be ∼2 kcal/mol lower when Asp257 rather than Asp385 is protonated. This result provides an important piece of missing information for assessing the protonation state preference of the catalytic dyad apart from the difference in p*K*_a_ of the two aspartates determined in previous studies. We further find that the disparity in the reaction barrier most likely arises from the stabilization difference in the residues close to the chemical reaction center, particularly for the Arg269 located in PS1 subunit of γ-secretase.

Numerous computational works of γ-secretase MD studies with Asp257-protonated,^18,19,22,71^ Asp385-protonated,^57,72,73^ both unprotonated,^74^ or both protonated^58,75,76^ have so far been conducted. The pH replica exchange molecular dynamics (pH-REMD) simulations by Guzmán-Ocampo et al. revealed that upon binding of the substrate to the active site of γ-secretase, the Asp385 is more likely to be protonated compared to Asp257 due to the p*K*_a_ of 385 being 6.6 and that of 257 being 3.65. The p*K*_a_ difference between the Asp257 and Asp385 is ∼3 units, corresponding to a free energy difference of about 4 kcal/mol.^21^ Our finding that Asp257 is most likely protonated for catalysis does not exclude the possibility that the other protonation state may also be adopted transiently (or even mostly). The protonation conclusions obtained by various works in different conformational states are inconsistent; perhaps the enzyme is not the only protonation state in the whole catalytic cycle. At least when the catalysis reaction is really initiated, Asp257 will be protonated. Structural data from X-ray and neutron diffraction reveal that one of the catalytic aspartic residues in aspartate protease is protonated and the other is unprotonated.^16,17,77,78^ Thus, it is worth noting that the issue of the protonation state of catalytic dyad in the γ-secretase will eventually be settled by the neutron diffraction experiments technique. Therefore, our results provide a crucial layer of details regarding the catalytic mechanism of γ-secretase, which could be helpful for the development of a therapeutic approach targeting this enzyme.

## Supporting Information Available

Superimposed structures of the latter two types of the active site conformation from unbiased extensive simulations (Figure S1); representative structures selected to build the hybrid QM/MM models (Figures S2 and S3); free energy landscape Δ*G*_SC_ distributed along two dihedrals (ϕ_V50_ and ψ_L49_) in the extensive unbiased simulations (Figure S4); sampled CV values from QM/MM metadynamics MD simulations (Figure S5); reconstructed 2D-PMF landscapes in different time intervals to determine the convergence of QM/MM umbrella sampling simulations (Figures S6–S10); summary of calculations at SCC-DFTB/CHARMM36m level from other types (Figures S11–S13); structure for the other gem-diol intermediate in both protonated states (Figure S14); small-molecule model of the active site used in benchmarking calculations in both protonated states (Figure S15); Gibbs free energy profiles in different calculation levels in both protonated states (Figure S16); values for two CVs in two calculated models in both protonated states (Figure S17); different gem-diol intermediates in the small model (Figure S18); details for CVs in QM/MM MD simulations (Table S1); details of parameters in QM/MM metadynamics MD simulations (Table S2); benchmarking energies for the small-molecule gas-phase system in both protonated states (Table S3); and relative energies for the different gem-diol intermediates to examine the stability of unexpected gem-diol using the small-molecule model (Table S4). (PDF)
Simulation parameter files and the structures of the reactants, transition states, and gem-diol intermediates in different protonation states. (ZIP)
