# Supplementary material for: Selective Protonation of Catalytic Dyad for γ-Secretase-Mediated Hydrolysis Revealed by Multiscale Simulations
Source: J Phys Chem B. 2024 Nov 7;128(46):11345–58. doi: 10.1021/acs.jpcb.4c04085 (PMC11586911; doi:10.1021/acs.jpcb.4c04085)
Supplement: Supplementary file 1 — jp4c04085_si_001.pdf [file jp4c04085_si_001.pdf]

Supporting Information

Selective Protonation of Catalytic Dyad for γ-Secretase-Mediated Hydrolysis Revealed by Multiscale Simulations

Bohua Wu[a], Shu Li[b], Wei Han[a,c,d,*]

[a] *State Key Laboratory of Chemical Oncogenomics, Guangdong Provincial Key Laboratory of Chemical Genomics, School of Chemical Biology and Biotechnology, Peking University Shenzhen Graduate School, Shenzhen, 518055, China*

[b] *Centre for Artificial Intelligence Driven Drug Discovery, Faculty of Applied Sciences, Macao Polytechnic University, Macao, 999078, China*

[c] *Department of Chemistry, Faculty of Science, Hong Kong Baptist University, Hong Kong SAR, China*

[d] *Institute of Chemical Biology, Shenzhen Bay Laboratory, Shenzhen 518132, China*

* Email: hanw_chem@hkbu.edu.hk

Contents

**Figure S1**	The superimposed structures of the latter two types of protonation states from unbiased extensive simulations in both protonated systems.
**Figure S2**	The representative structure used to perform QM/MM calculations.
**Figure S3**	The representative structure of other types used to perform QM/MM calculations.
**Figure S4**	The free energy landscape ΔG_{SC} distributed along two dihedrals (ϕ_{V50} and ψ_{L49}) in the extensive unbiased simulations.
**Figure S5**	The sampled CV values from QM/MM metadynamics MD simulations.
**Figure S6**	The convergence examination in the protonation of Asp257 when the reactant state is from Asp257i_385i.
**Figure S7**	The convergence examination in the protonation of Asp385 when the reactant state is from Asp385i_257i.
**Figure S8**	The convergence examination in the protonation of Asp257 when the reactant state is from Asp257no_385i.
**Figure S9**	The convergence examination in the protonation of Asp385 when the reactant state is from Asp385no_257o.
**Figure S10**	The convergence examination in the protonation of Asp385 when the reactant state is from Asp385i_257o.
**Figure S11**	The reaction calculated at the SCC-DFTB/CHARMM36m level from the Asp257no_385i with the D257-protonated system.
**Figure S12**	The reaction calculated at the SCC-DFTB/CHARMM36m level from the Asp385no_257o with the D385-protonated system.
**Figure S13**	The reaction calculated at the SCC-DFTB/CHARMM36m level from the Asp385i_257o with the D385-protonated system.
**Figure S14**	The structures for the other gem-diol intermediate in both protonated states.
**Figure S15**	The small molecule model used in benchmarking calculations.
**Figure S16**	The Gibbs free energy profiles in different calculation levels.
**Figure S17**	The values for two CVs in two calculated models in both protonated states.
**Figure S18**	The different gem-diol intermediates in the small model.
**Table S1**	The details for CVs in QM/MM MD simulations.
**Table S2**	The details of parameters in QMMM metadynamics MD simulations.
**Table S3**	Benchmarking energies for the small molecule gas-phase system in both protonation states.
**Table S4**	Relative energies for the different gem-diol intermediates.

MM MD simulations

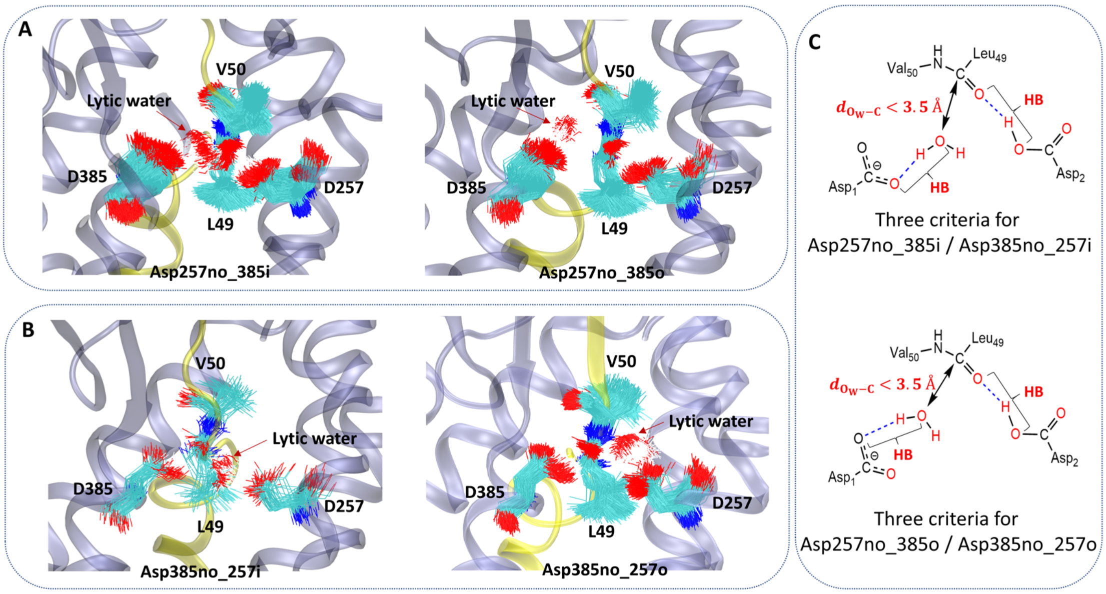

Figure S1. The superimposed active structures of the latter two types in the D257-protonated system (A) and D385-protonated system (B). (C) Three criteria to determine Asp257no_385i/Asp385no_257i (upper) and Asp257no_385o/Asp385no_257o (bottom) in both protonation states.

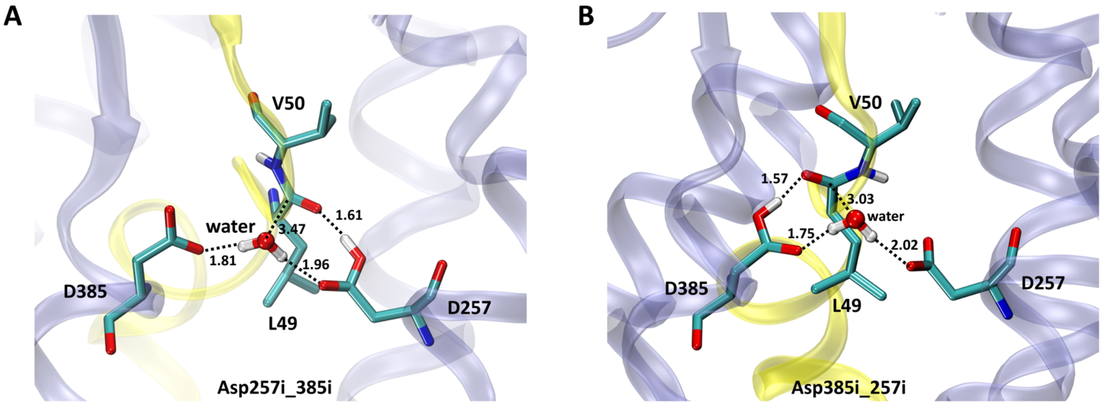

Figure S2. The representative structures of Asp257i_385i for the D257-protonated system (A) and Asp385i_257i for the D257-protonated system (B). The labeled distances are in Å. These representative structures are used to performed QM/MM MD.

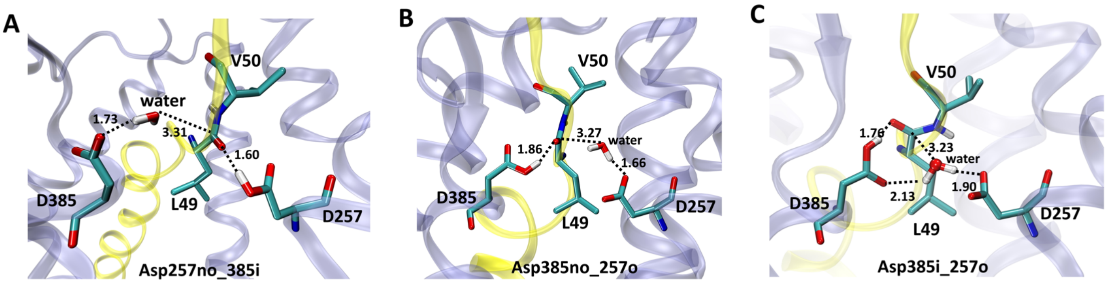

Figure S3. The representative structures of Asp257no_385i for the D257-protonated system (A), Asp385no_257o for the D385-protonated system (B) and Asp385i_257o for the D385-protonated system (C). The labeled distances are in Å. The representative structures are used to performed QM/MM MD.

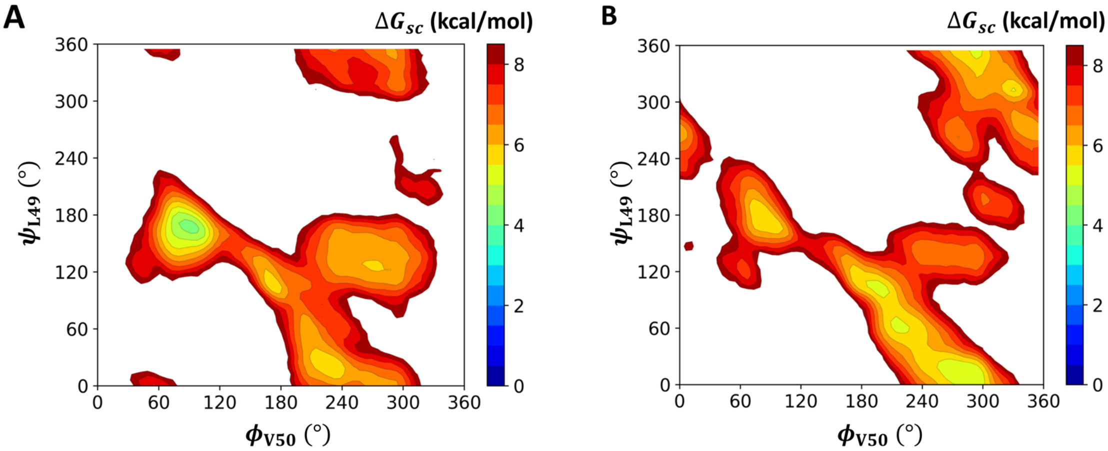

Figure S4. The free energy landscape $\Delta G_{\rm SC}$ distributed along two dihedrals ($\phi_{\rm V50}$ and $\psi_{\rm L49}$) in the extensive unbiased simulations with the D257-protonated state (A) and D385-protonated state (B). The $\Delta G_{\rm SC}$ is defined as $-RT\ln P(\phi_{\rm V50}, \psi_{\rm L49})$.

QM/MM MD simulations

Table S1. The details for CVs in QM/MM MD simulations

**Simulation system**	**Collective variables**	**Definition**
D257-P	CV1: $d_{\mathrm{O1-H_{Asp257}}} - d_{\mathrm{(O-H)_{Asp257}}}$<br>CV2: $d_{\mathrm{O_w-H_w}} - d_{\mathrm{O_W-C1}}$	CV1: proton transfer between Asp257 and Leu49<br>CV2: nucleophilic attack by the activated water
D385-P	CV1: $d_{\mathrm{O1-H_{Asp385}}} - d_{\mathrm{(O-H)_{Asp385}}}$<br>CV2: $d_{\mathrm{O_w-H_w}} - d_{\mathrm{O_W-C1}}$	CV1: proton transfer between Asp385 and Leu49<br>CV2: nucleophilic attack by the activated water

Table S2. The details of parameters in QMMM metadynamics MD simulations

**Simulation system**	**Bias -factor**	**Number of CVs**	**Gaussian width**	**Gaussian height**	**T (K)**	**Deposited frequency**
D257-P	10	2	0.3,0.5	2.0	303.15	100
D385-P	20	2	0.3,0.5	2.0	303.15	100

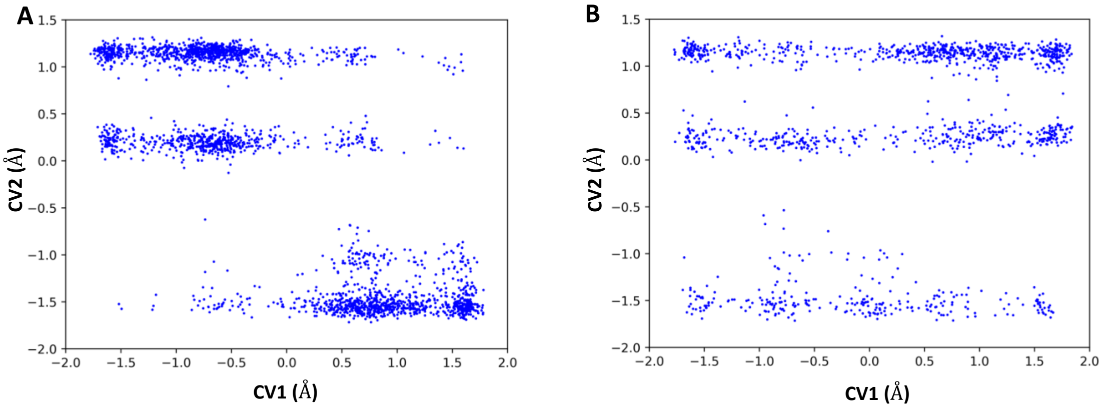

Figure S5. The sampled CV values from QM/MM metadynamics MD simulations with the D257-protonated state (A) and D385-protonated state (B). The results in both figures obtained in the presence of biasing potentials.

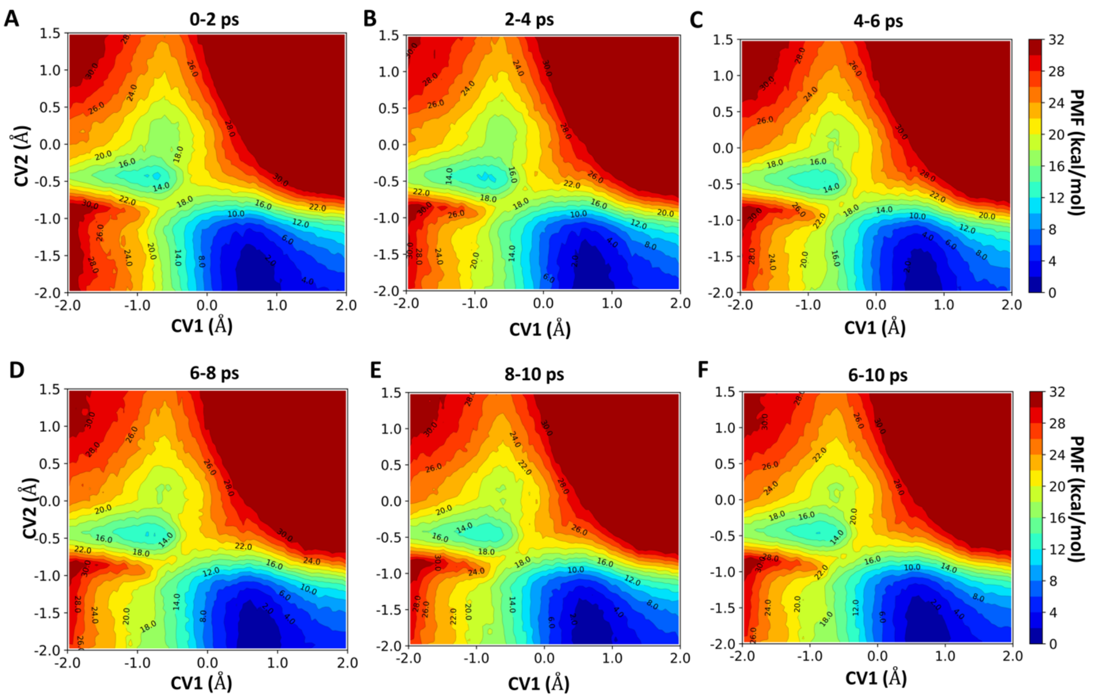

Figure S6. The reconstructed 2D-PMF in different time intervals at SCC-DFTB/CHARMM36m for the formation of gem-diol with the D257-protonated state. The reactant state was from the Asp257i_385i of active site conformation.

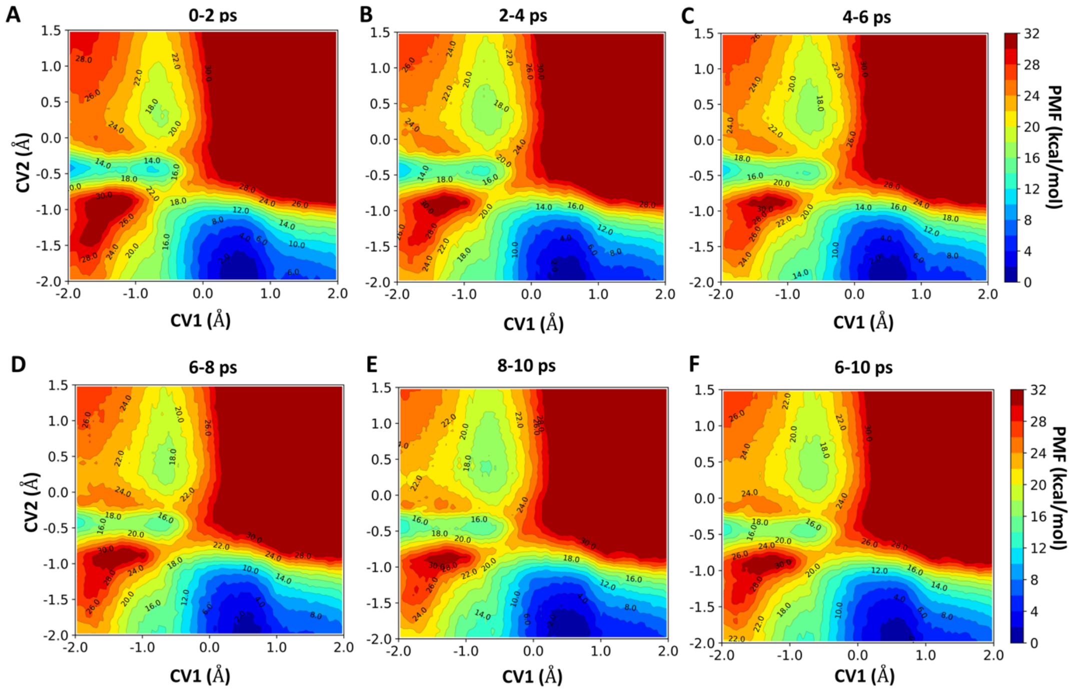

Figure S7. The reconstructed 2D-PMF in different time intervals at SCC-DFTB/CHARMM36m for the formation of gem-diol with the D385-protonated state. The reactant state was from the Asp385i_257i of active site conformation.

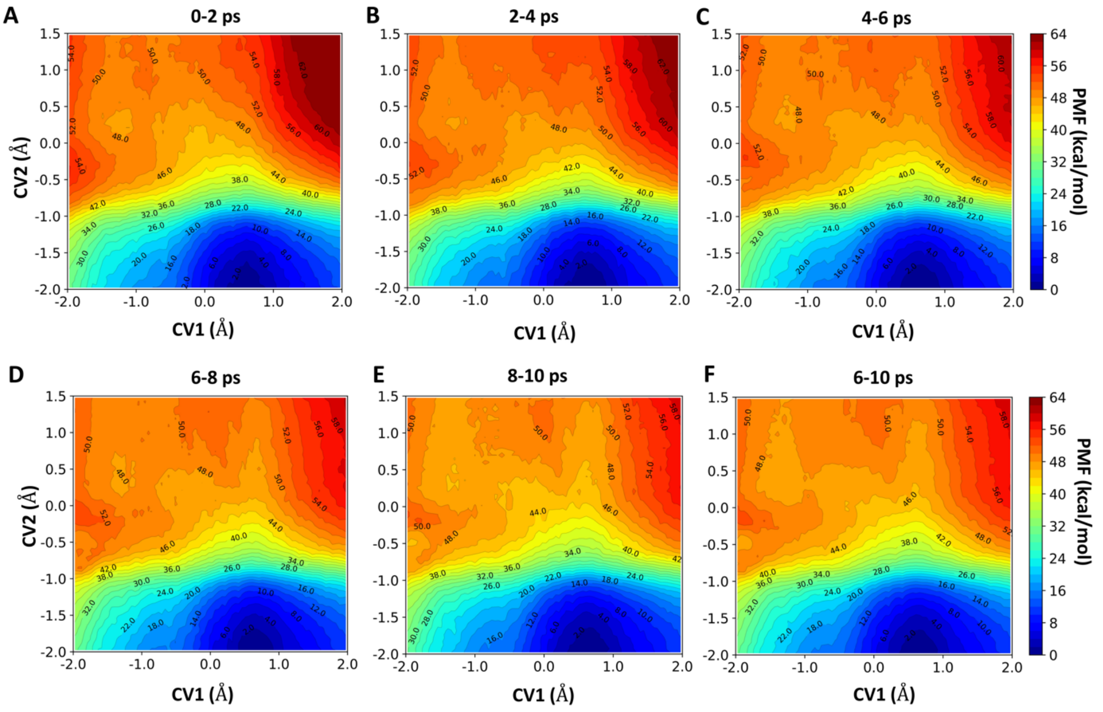

Figure S8. The reconstructed 2D-PMF in different time intervals at SCC-DFTB/CHARMM36m for the formation of gem-diol with the D257-protonated state. The reactant state was from the Asp257no_385i of active site conformation.

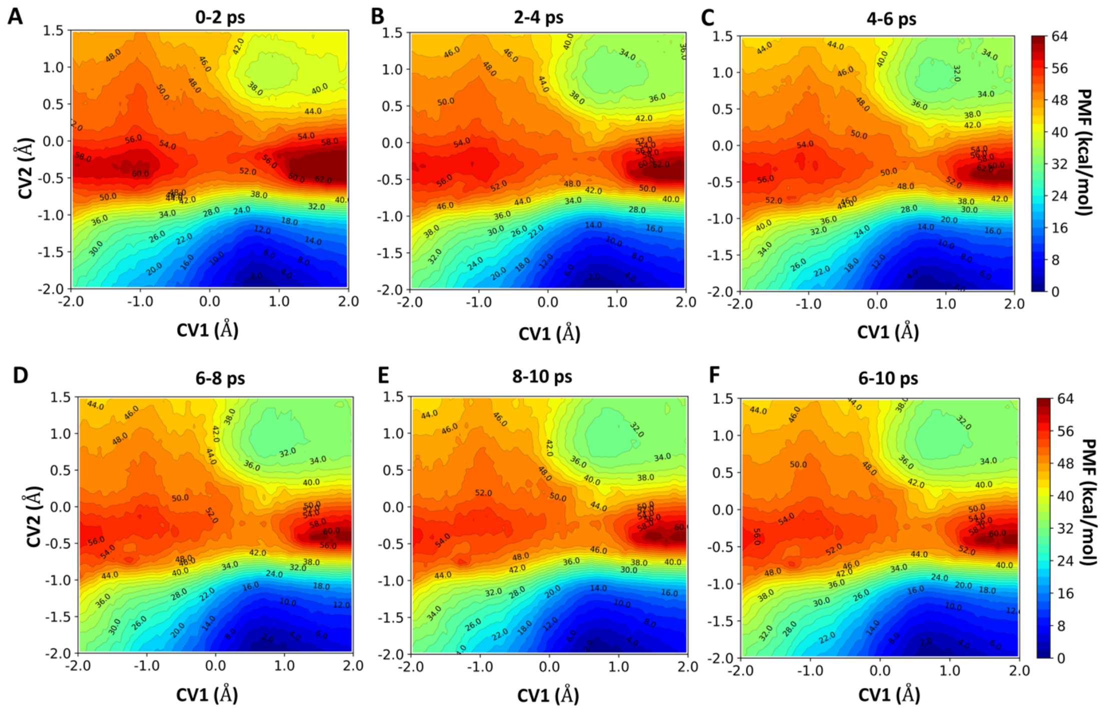

Figure S9. The reconstructed 2D-PMF in different time intervals at SCC-DFTB/CHARMM36m for the formation of gem-diol with the D385-protonated state. The reactant state was from the Asp385no_257o of active site conformation.

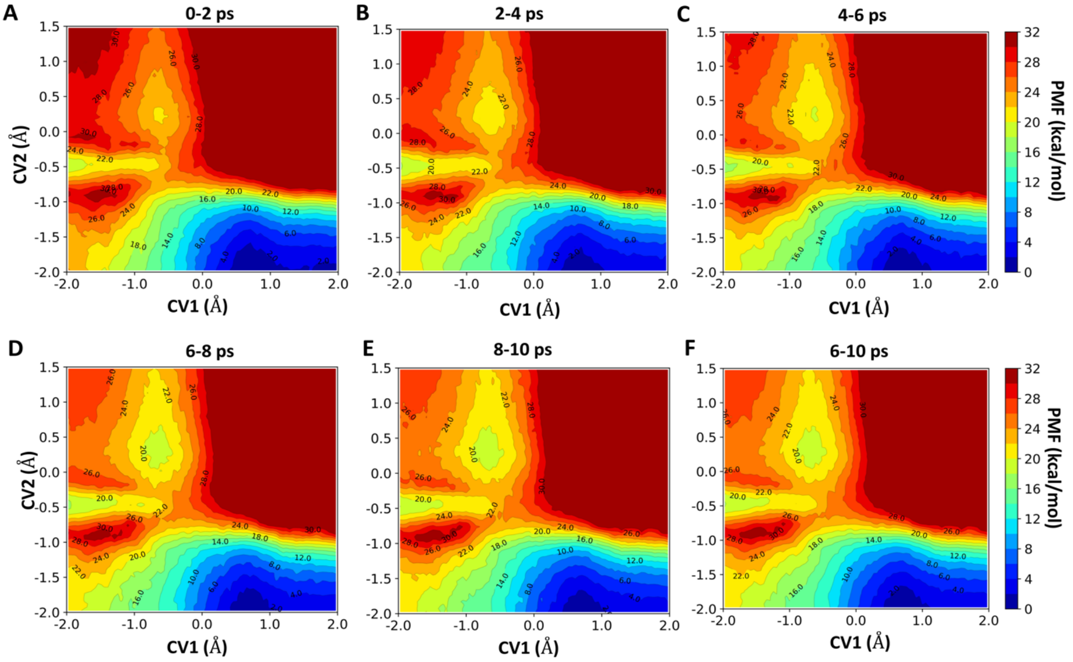

Figure S10. The reconstructed 2D-PMF in different time intervals at SCC-DFTB/CHARMM36m for the formation of gem-diol with the D385-protonated state. The reactant state was from the Asp385i_257o of active site conformation.

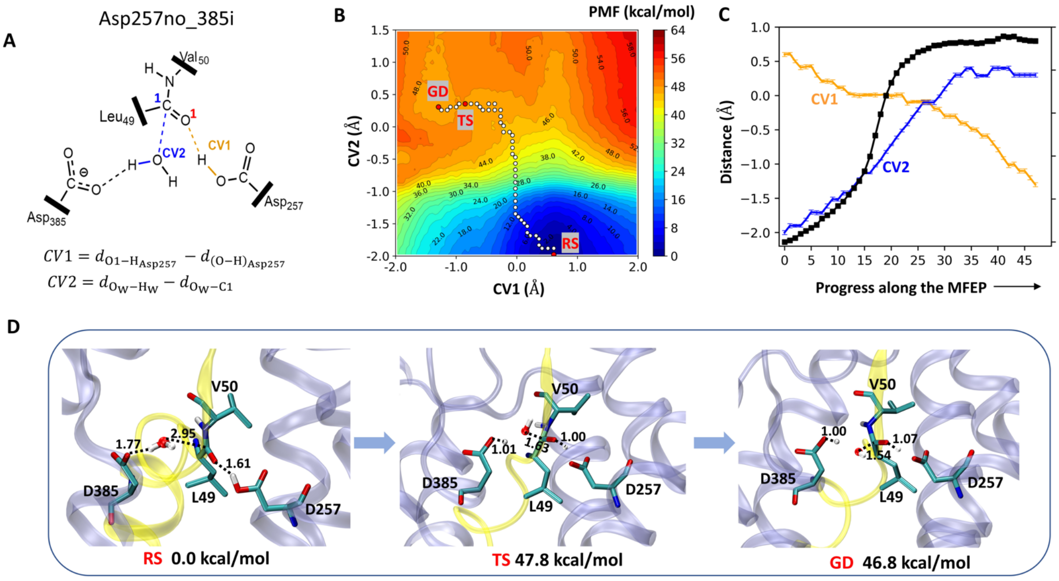

Figure S11. Summary of the formation of gem-diol catalyzed by γ-secretase calculated at the SCC-DFTB/CHARMM36m level with the D257-protonated system. The reactant state was from the Asp257no_385i of active site conformation. (A) Two CVs were used to conduct the umbrella sampling. (B) The reconstructed reaction 2D-PMF along defined two CVs. The white and red circles show the free energy profile along the MFEP. RS, TS and GD denote the reactant state, the transition state and the gem-diol intermediate state, respectively. (C) Values for the free energy profile (black line), CV1 (orange line) and CV2 (blue line) along the MFEP. The error bars are marked in every point along the MFEP. (D) Structures of the key states in the gem-diol formation catalyzed by γ-secretase. The labeled distances are in Å. The two CVs values for three structures:

$CV1 = 0.6$ Å / $CV2 = -2.0$ Å in RS, $CV1 = -0.9$ Å / $CV2 = 0.4$ Å in TS and $CV1 = -1.3$ Å / $CV2 = 0.3$ Å in GD.

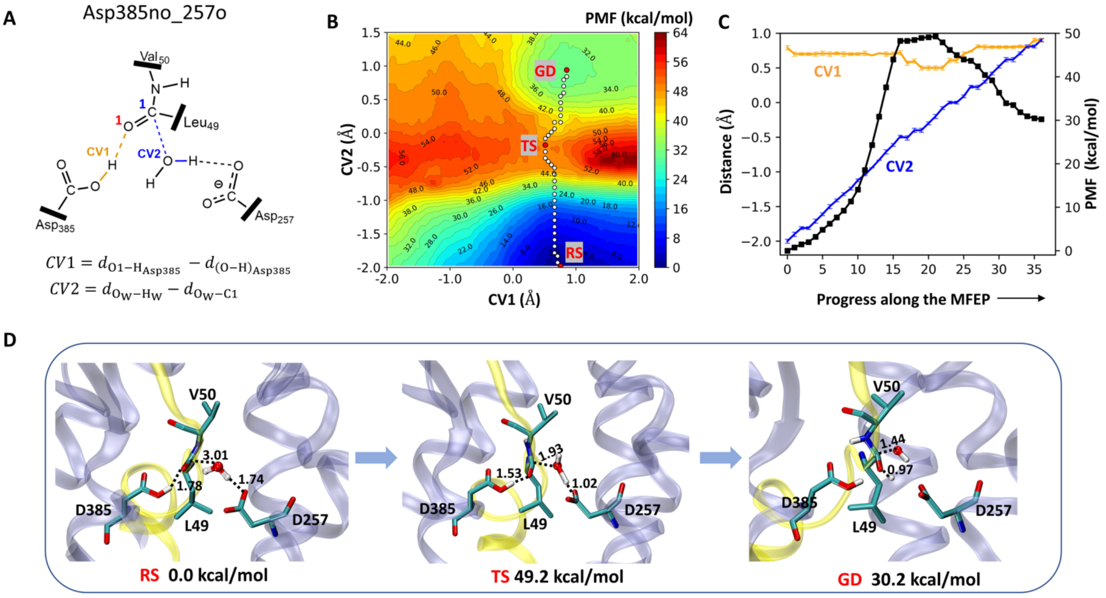

Figure S12. Summary of the formation of gem-diol catalyzed by γ-secretase calculated at the SCC-DFTB/CHARMM36m level with the D385-protonated system. The reactant state was from the Asp385no_257o of active site conformation. (A) Two CVs were used to conduct the umbrella sampling. (B) The reconstructed reaction 2D-PMF along defined two CVs. The white and red circles show the free energy profile along the MFEP. RS, TS and GD denote the reactant state, the transition state and the gem-diol intermediate state, respectively. (C) Values for the free energy profile (black line), CV1 (orange line) and CV2 (blue line) along the MFEP. The error bars are marked in every point along the MFEP. (D) Structures of the key states in the gem-diol formation catalyzed by γ-secretase. The labeled distances are in Å. The two CVs values for three structures: $CV1 = 0.8$ Å / $CV2 = -2.0$ Å in RS, $CV1 = 0.5$ Å / $CV2 = -0.2$ Å in TS and $CV1 = 0.8$ Å / $CV2 = 1.0$ Å in GD.

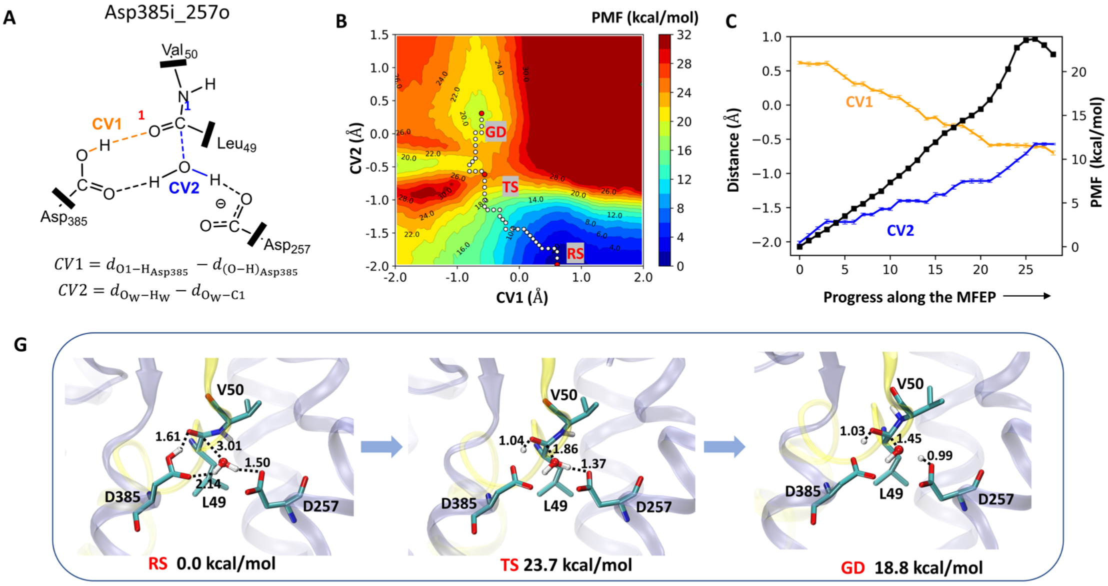

Figure S13. Summary of the formation of gem-diol catalyzed by γ-secretase calculated at the SCC-DFTB/CHARMM36m level with the D385-protonated system. The reactant state was from the Asp385i_257o of active site conformation. (A) Two CVs were used to conduct the umbrella sampling. (B) The reconstructed reaction 2D-PMF along defined two CVs. The white and red circles show the free energy profile along the MFEP. RS, TS and GD denote the reactant state, the transition state and the gem-diol intermediate state, respectively. (C) Values for the reaction free energy profile (black line), CV1 (orange line) and CV2 (blue line) along the MFEP. The error bars are marked in every point along the MFEP. (G) Structures of the key states in the gem-diol formation catalyzed by γ-secretase. The labeled distances are in Å. The two CVs values for three structures: $CV1 = 0.6$ Å / $CV2 = -2.0$ Å in RS, $CV1 = -0.5$ Å / $CV2 = -0.7$ Å in TS and $CV1 = -0.7$ Å / $CV2 = 0.4$ Å in GD.

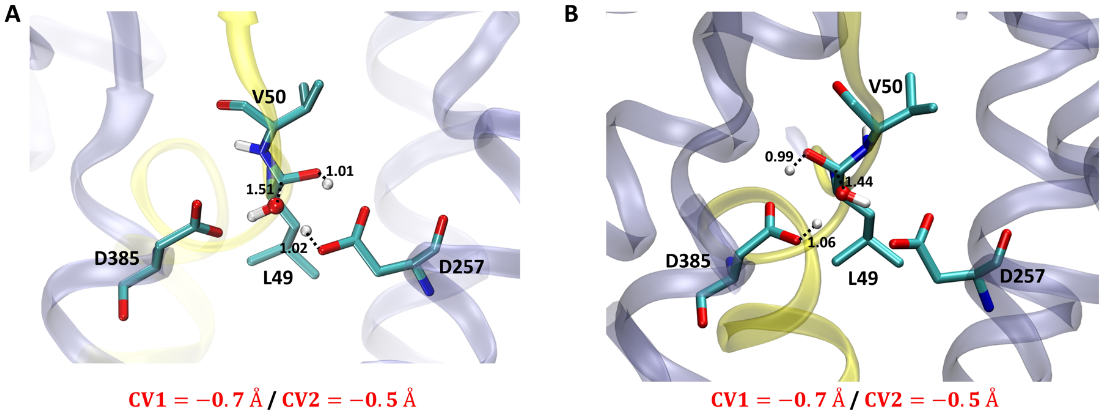

Figure S14. The other unexpected gem-diol intermediate (GD’) with the D257-protonated state (A) and the D385-protonated state (B). The labeled distances are in Å.

Benchmarking

The known benchmark method on small molecule model has been reported in previous work.[1] To benchmark the performance of SCC-DFTB, we performed transition state optimization for a representative small molecule model in gas-phase and calculation a potential energy surface using a popular hybrid DFT functional, B3LYP-D3, with single point energy corrections (B3LYP-D3/def2-TZVP). The small molecule model used in benchmarking is presented in Figure S15. The gas-phase model included L49, V50, an activated water molecule and the side chains of D257 and D385 was taken from the both protonated systems. Transition state (TS) was first optimized using Gaussian09 on the B3LYP-D3/6-31G(d,p) level. The ultrafine integration grid was used in all calculations (int=ultrafine). TS was validated by visual inspection and normal model calculation (only one imaginary frequency at -195.43 in the D257-protonated state and at -175.83 in the D385-protonated state corresponding to the reaction coordinates). The optimization and frequency output are included in the Supporting Information ZIP file. The reactant (RC) and tetrahedral gem-diol intermediate (GD) were obtained with IRC calculations in both directions starting from the optimized TS, and the final IRC endpoints were further optimized on the same level as the TS. Single-point energies were calculated on the higher B3LYP-D3/def2-TZVP level, as well as using SCC-DFTB.

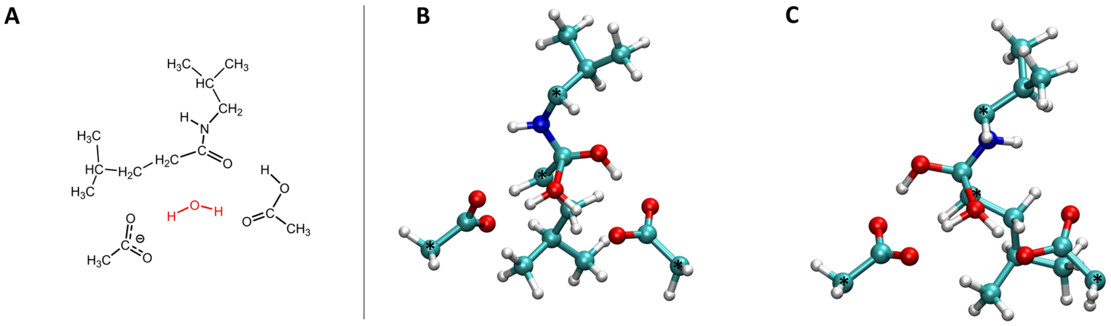

Figure S15. (A) The small molecule model of the active site used in benchmarking calculations. The initial structure of transition state taken from the QM/MM simulation in D257-protonated system (B) and the D385-protonated (C). The small model includes Asp257 and Asp385 truncated at the β carbon, an activated water, L49 and V50 truncated at the α carbon. The black stars denote the truncated site.

Table S3. Benchmarking energies for the small molecule gas-phase system

System	Method	RS CV1 (Å)	RS CV2 (Å)	TS CV1 (Å)	TS CV2 (Å)	*ΔG* (kcal/mol)
D257-P	B3LYP-D3/6-31G**	0.47	-2.2	-0.46	-0.72	16.9
	B3LYP-D3/def2-TZVP[a]	_	_	_	_	20.7
	SCC-DFTB[a,b]	_	_	_	_	11.5
	SCC-DFTB/CHARMM36m[c]	0.70	-1.98	-0.41	-0.76	19.8
D385-P	B3LYP-D3/6-31G**	0.41	-2.14	-0.54	-0.70	17.4
	B3LYP-D3/def2-TZVP[a]	_	_	_	_	20.3
	SCC-DFTB[a,b]	_	_	_	_	11.4
	SCC-DFTB/CHARMM36m[c]	0.61	-1.97	-0.46	-0.71	22.1

[a]The energy difference from single-point energies on the RS and TI optimized at B3LYP-D3/6-31G(d,p) level.

[b]The could not be optimized in the gas-phase with SCC-DFTB as no saddle point was indicated between RS minimum and the gem-diol tetrahedral intermediate.

[c]The approximate RS and TS locations on the QM/MM 2D-PMF.

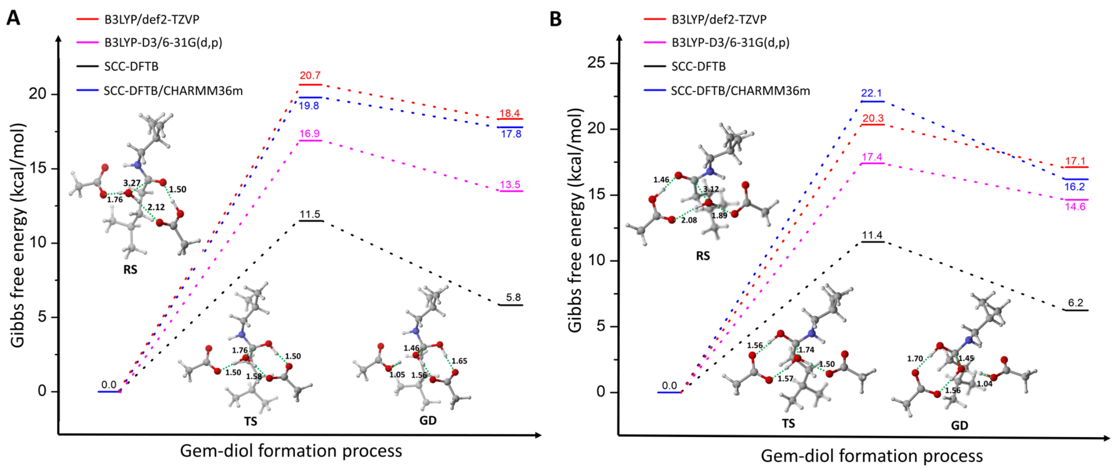

Figure S16. The Gibbs free energy profiles in different calculation levels with the D257-protonated state (A) and D385-protonated state (B). Optimized structures involved in the gem-diol formation process were at the B3LYP-D3/6-31G(d,p) level.

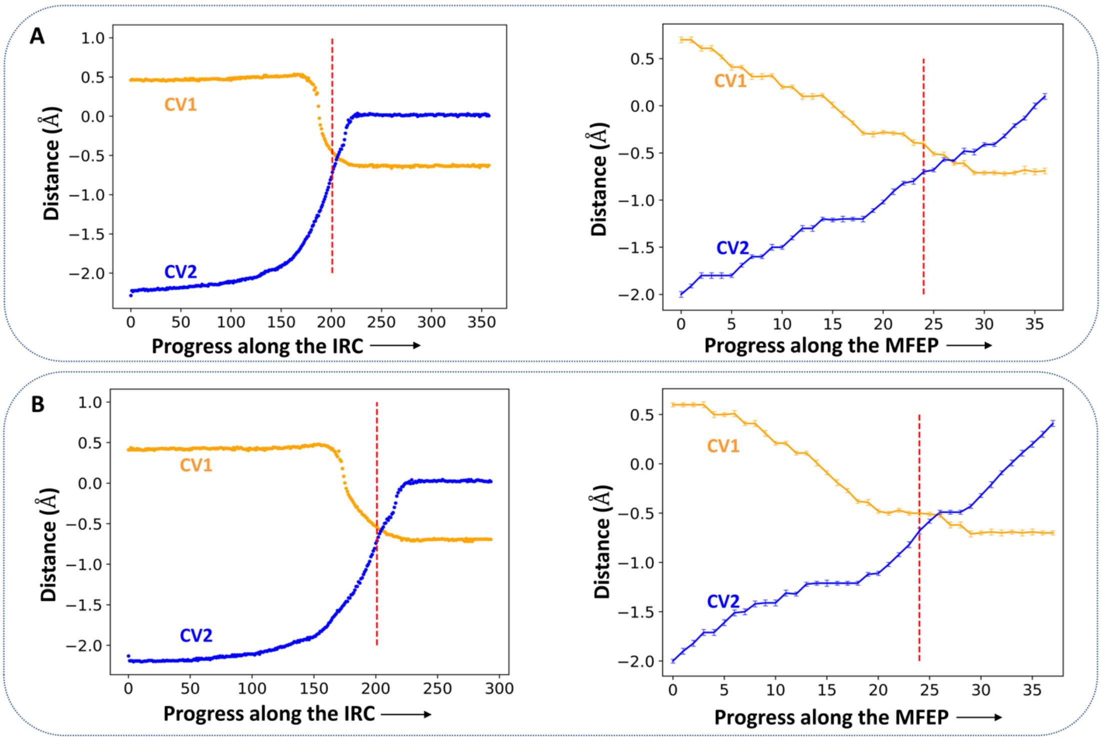

Figure S17. The values for two CVs along the IRC (left, from the QM calculation) and MFEP (right, from the QM/MM MD simulations) with D257-protonated state (A) and D385-protonated state (B). The dashed red lines denote the position of transition state in both models in (A) and (B).

Table S4. Relative energies for the different gem-diol intermediates [a]

**System**	**Method**	**$\Delta E_{GD'}$ (kcal/mol)**	**ΔE_{GD} (kcal/mol)**	**$\Delta\Delta E$ ($E_{GD'}$-E_{GD}) (kcal/mol)**
D257-P	B3LYP-D3/6-31G**	0	-4.40	4.40
	SCC-DFTB	0	-13.30	13.30
D385-P	B3LYP-D3/6-31G**	0	-6.18	6.18
	SCC-DFTB	0	-12.03	12.03

[a] Relative energies are the single-point energies from the optimization process for the two protonation states. GD' and GD corresponding to the optimized starting and endpoint structure for the two protonation states, respectively. The single-point energy of unexpected gem-diol (GD') is as the reference.

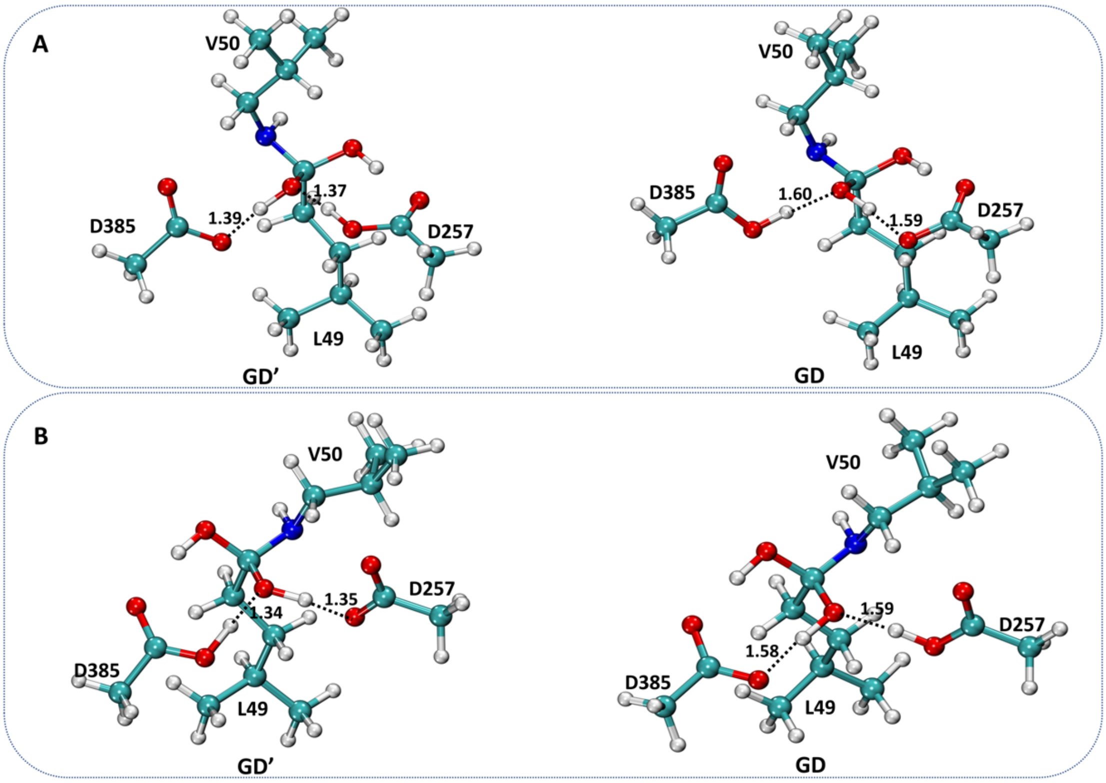

Figure S18. The expected gem-diol (GD) and unexpected gem-diol (GD’) with the D257-protonated state (A) and D385-protonated state (B). Structures displayed were optimized at the B3LYP-D3/6-31G(d,p) level.

References

1. Hirvonen, V. H. A.; Weizmann, T. M.; Mulholland, A. J.; Spencer, J.; van der Kamp, M. W., Multiscale Simulations Identify Origins of Differential Carbapenem Hydrolysis by the OXA-48 beta-Lactamase. *Acs Catalysis* **2022,** *12* (8), 4534-4544.
